# Extensive homoeologous genome exchanges in allopolyploid crops revealed by mRNAseq‐based visualization

**DOI:** 10.1111/pbi.12657

**Published:** 2016-12-06

**Authors:** Zhesi He, Lihong Wang, Andrea L. Harper, Lenka Havlickova, Akshay K. Pradhan, Isobel A. P. Parkin, Ian Bancroft

**Affiliations:** ^1^ Department of Biology University of York Heslington York UK; ^2^ Department of Genetics and Centre for Genetic Manipulation of Crop Plants University of Delhi New Delhi India; ^3^ Agriculture and Agri‐Food Canada Saskatoon SK Canada

**Keywords:** crop genomes, genome structural evolution, mRNAseq

## Abstract

Polyploidy, the possession of multiple sets of chromosomes, has been a predominant factor in the evolution and success of the angiosperms. Although artificially formed allopolyploids show a high rate of genome rearrangement, the genomes of cultivars and germplasm used for crop breeding were assumed stable and genome structural variation under the artificial selection process of commercial breeding has remained little studied. Here, we show, using a repurposed visualization method based on transcriptome sequence data, that genome structural rearrangement occurs frequently in varieties of three polyploid crops (oilseed rape, mustard rape and bread wheat), meaning that the extent of genome structural variation present in commercial crops is much higher than expected. Exchanges were found to occur most frequently where homoeologous chromosome segments are collinear to telomeres and in material produced as doubled haploids. The new insights into genome structural evolution enable us to reinterpret the results of recent studies and implicate homoeologous exchanges, not deletions, as being responsible for variation controlling important seed quality traits in rapeseed. Having begun to identify the extent of genome structural variation in polyploid crops, we can envisage new strategies for the global challenge of broadening crop genetic diversity and accelerating adaptation, such as the molecular identification and selection of genome deletions or duplications encompassing genes with trait‐controlling dosage effects.

## Introduction

Polyploid organisms have multiple sets of chromosomes, and genome studies indicate that the evolutionary flexibility endowed by polyploidy has shaped the genomes of most if not all eukaryotes (Comai, 2005). Stable polyploidy occurs in fish and frogs (Gregory and Mable, 2005), although plants offer the best systems for its study as this is where it is most widespread. Indeed, it has been considered a predominant factor in the evolution and success of the angiosperms (Leitch and Bennett, 1997; Wendel, 2000). As part of the genome stabilization process termed ‘diploidization’ (Hillier *et al*., 2004; Wang *et al*., 2005), newly formed polyploid genomes undergo rapid structural evolution, including gene copy number variation (CNV) (Adams and Wendel, 2005). As CNVs are frequently associated with genetic traits (Beckmann *et al*., 2007), they are likely to be of crucial importance to crop science as many important crops (e.g. bread wheat, cotton, soybean, potato and rapeseed) are recently formed polyploids. Inferring the mechanisms involved in the diploidization process by comparative genomics of extant species has become a key aim of plant genomics. However, the lack of cost‐effective analysis tools means that there has been relatively little analysis of genome structural variation in commercial varieties of polyploid crops.

Bread wheat (*Triticum aestivum*) is an allohexaploid comprising three genomes: A, B and D (Chantret *et al*., 2005). Less than 800 000 years ago, a hybridization between the A genome progenitor, *Triticum urartu,* and the B genome progenitor (a close relative of *Aegilops speltoides*) formed the AABB allotetraploid emmer wheat *T. turgidum*. Finally, less than 400 000 years ago, hybridization between emmer wheat and the D genome progenitor, *Aegilops tauschii* (Marcussen *et al*., 2014), formed the AABBDD allohexaploid bread wheat, *T. aestivum*.

The cultivated *Brassica* species are the group of crops most closely related to *Arabidopsis thaliana*, which was the first plant for which a high‐quality genome sequence was available (The Arabidopsis Genome Initiative, 2000). The species *Brassica rapa* and *Brassica oleracea*, which contain the *Brassica* A and C genomes, respectively, last shared a common ancestor *ca*. 3.7 Mya (Inaba and Nishio, 2002). *Brassica napus* is an allopolyploid, arising from the hybridization of A and C genome progenitors (U.N., 1935), and the related (homoeologous) regions of the genomes are clearly discernible (Bancroft *et al*., 2015). A diverse range of *B. napus* crop types have already been developed, including oilseed rape, fodder types, leafy vegetables (kale types) and root vegetables (swede or rutabaga), underlining the phenotypic plasticity of recently formed polyploids such as *B. napus*. Analysis of the genome sequence reported for an oilseed rape *B. napus* variety (Chalhoub *et al*., 2014) provided clear evidence for homoeologous exchanges (HEs). These were characterized by the loss of a chromosomal region that was replaced by a duplicate copy of the corresponding homoeologous region of the other genome. This indicates that HEs occurred at some stage in the formation of the particular crop variety sequenced.

Genome resequencing can be used to study HEs. Indeed, this approach was used to identify putative HEs in six varieties of *B. napus* crops (Chalhoub *et al*., 2014). Even using NGS sequencing technology, however, this is an expensive approach. In contrast, transcriptome‐based studies using mRNAseq are particularly rapid and cost‐effective, with a suite of methodologies developed and applied in *B. napus* for SNP discovery (Trick *et al*., 2009), linkage mapping and genome characterization (Bancroft *et al*., 2011), transcript quantification (Higgins *et al*., 2012) and association genetics (Harper *et al*., 2012).

In this study, we exploited a method we developed recently for assessing genome dominance in polyploid species (Harper *et al*., 2016) to visualize HEs in allopolyploid crops and assess the extent to which these occur in cultivars.

## Results

### mRNAseq‐based visualization of homoeologous genome exchanges in *Brassica napus*

**Figure 1:**
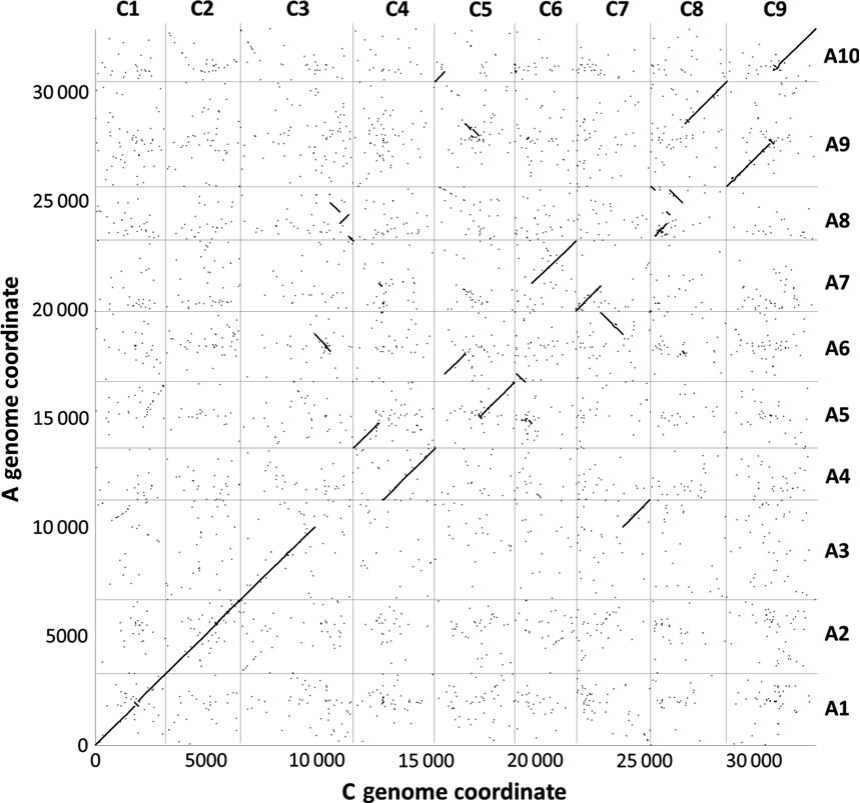
Homoeology relationships between *Brassica* A and C genomes. 32 904 pairs of homoeologous genes in the *Brassica* A and C genomes are plotted by their order in the respective genomes.

**Figure 2:**
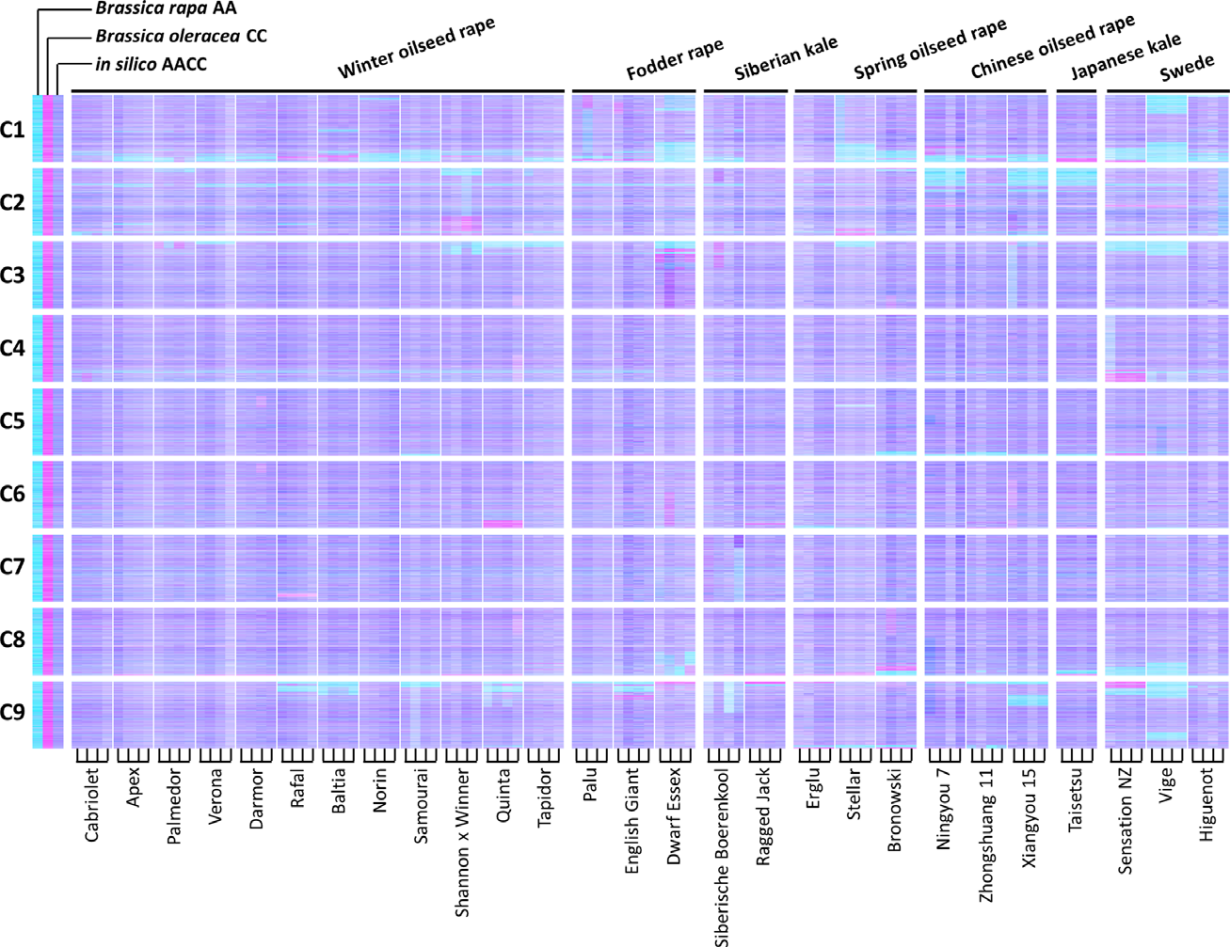
Visualization of homoeologous genome exchanges in *Brassica napus* using Transcriptome Display Tile Plots based on mRNAseq data. The relative transcript abundance of A and C genome homoeologous gene pairs is represented in CMYK colour space, with cyan component representing transcript abundance of the *Brassica* A genome copy and magenta component representing transcript abundance of the *Brassica* C genome copy. The pairs are plotted in *Brassica* C genome order (chromosomes denoted C1 to C9) for four biological replicates of each of 27 accessions of *B. napus* and controls comprising parental species and their *in silico* combination.

**Figure 3:**
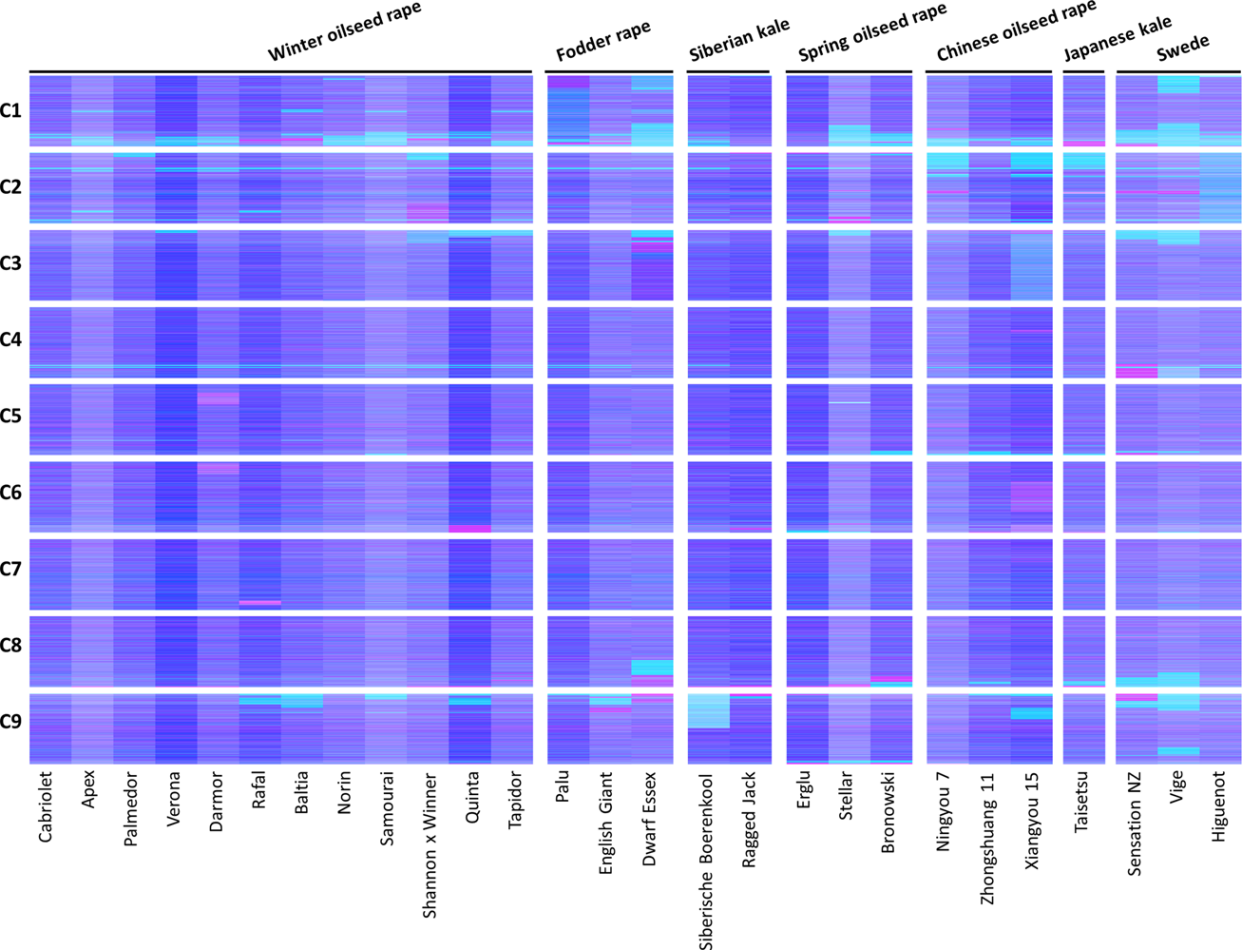
Visualization of homoeologous genome exchanges based on DNA resequencing. The relative redundancy of coverage of A and C genome homoeologous gene pairs is represented in CMYK colour space, with cyan component representing coverage of the *Brassica* A genome copy and magenta component representing coverage of the *Brassica* C genome copy. The pairs are plotted in Brassica C genome order (chromosomes denoted C1 to C9).

We produced leaf mRNAseq data from a panel of 27 *B. napus* varieties, in four biological replicates, and quantified gene expression by mapping the reads to an ordered pan‐transcriptome resource that ensured correct allocation of *B. napus* gene sequences to either the *Brassica* A or C genome (He *et al*., 2015) (Data S1). We identified homoeologous gene pairs from the ordered pan‐transcriptome resources. The result was a set of 32 904 homoeologous pairs (Data S2), which define the homoeology relationships between the genomes as shown in Figure 1. Analysis of differential gene expression into the nine categories indicative of genome dosage changes as listed in Table 1 (Data S3, S4 and S5) revealed numerous clearly defined blocks in the genome where many nearby genes showed the same directionality of one genome over‐expressed with the other genome under‐expressed, suggestive of potential blocks of homoeologous exchange. To better visualize regions of the genome involved in homoeologous exchanges, we used Transcriptome Display Tile Plots (TDTPs), as used previously for visualizing genome dominance in wheat (Harper *et al*., 2016). This involves the following: (i) assigning quantitative transcript abundance for each member of each gene pair a value in CMYK colour space where the contributions from the A and C genome copies were coded to cyan and magenta channels, respectively; (ii) displaying the results using tile plots, with order based on the genome order of one member of the gene pair. The result is shown in Figure 2. Most of the genome displays blue, indicating approximately equal contributions to the transcriptome from each member of the homoeologous gene pairs. However, the predominant colour of several regions of the genome was cyan or magenta, indicative of HEs with increased copy number of the A and C genome segments, respectively. To confirm that the HEs identified were the result of structural exchanges rather than gene expression dominance effects, we undertook genome resequencing on one replicate from each of the 27 varieties and visualized the results in the equivalent manner. As shown in Figure 3, the results were identical, confirming the validity of using the more cost‐effective approach of undertaking such analyses based on mRNAseq rather than genome resequencing. We included in our experiment the swede variety Sensation NZ, which had been analysed previously by genome resequencing and mapping of reads to the Darmor‐*bzh* genome assemblies (Chalhoub *et al*., 2014). The TDTPs improved the analysis, showing clearer distinction between the balanced and exchanged regions. In addition, our approach identified an exchange affecting the bottom of chromosome C4 (and the homoeologous region of A4) that had not been detectable by mapping reads to the Darmor‐*bzh* genome assemblies (Chalhoub *et al*., 2014). The mRNAseq‐based analysis and TDTPs are very consistent between replicates and with genome resequencing results, presumably because of the relative nature of colour rendition within each homoeologue pair, meaning that biological replication is unnecessary for the identification of large HEs. All of the 27 cultivars analysed showed at least one HE, with some showing multiple exchanges.

**Table 1:** Categories of genome dosage changes

Expression of samples from one accession on A genome compared to all samples	Expression of samples from one accession on C genome compared to all samples	Gene dosage inference	Differential expression inference
High	High	Duplication A and C	One or both genome over‐expression
High	No change (null)	Duplication A	
No change (null)	High	Duplication C	
High	Low	Exchange C → A	One genome over‐expressed with the other genome under‐expressed relative to the mean of all samples
Low	High	Exchange A → C	
Low	No change (null)	Deletion A	One or both genome under‐expression
Low	Low	Deletion A and C	
No change (null)	Low	Deletion C	
No change (null)	No change (null)	No difference	No difference

*T*‐test analysis performed between the expression profiles of a single accession (with four replicated samples) on one genome (A or C) and mean of all samples. The expression profiles of each homoeologous CDS model can be allocated into nine categories of genome dosage inferences.

### Homoeologous genome exchanges in *Brassica napus* germplasm

**Figure 4:**
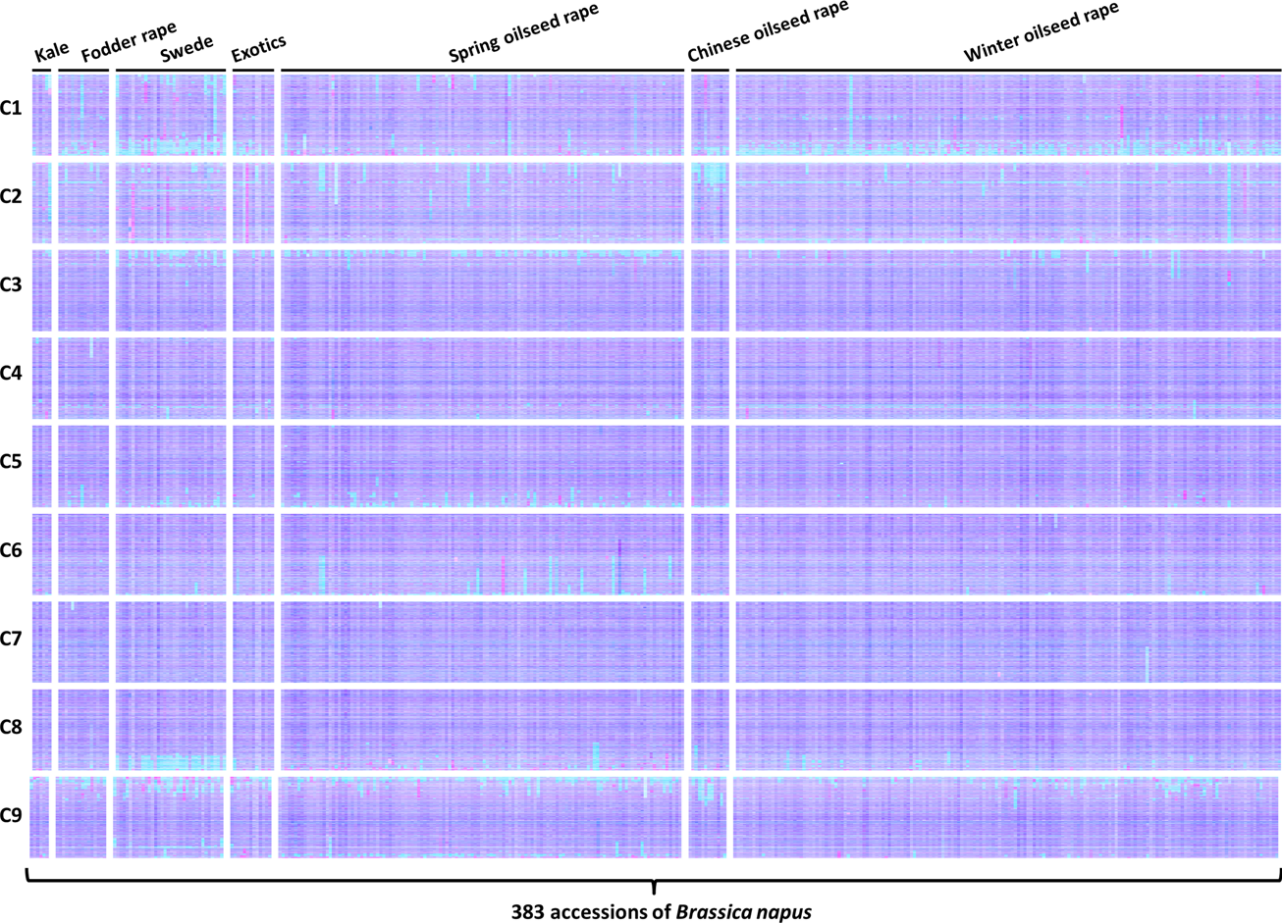
Visualization of homoeologous genome exchanges in *Brassica napus* RIPR diversity panel using Transcriptome Display Tile Plots based on mRNAseq data. The relative transcript abundance of A and C genome homoeologous gene pairs is represented in CMYK colour space, with cyan component representing transcript abundance of the *Brassica* A genome copy and magenta component representing transcript abundance of the *Brassica* C genome copy. The pairs are plotted in *Brassica* C genome order (chromosomes denoted C1 to C9).

To assess how representative the observations may be of the germplasm used by breeders, we produced TDTPs for a widely shared *B. napus* genetic diversity panel. This used recently produced mRNAseq data from 383 accessions comprising the ‘RIPR’ diversity panel (Data S6), which overlaps extensively with the ‘ASSYST’ diversity panel (Bus *et al*., 2011). The overview plots are shown in Figure 4. Remarkably, they show that all of the *B. napus* accessions examined contain identifiable segmental HEs. The sizes of these vary greatly. A few instances of whole chromosomes being duplicated or lost are observed, but this appears rare.

### Assessing the basis of variation for homoeologous genome exchanges in *Brassica* polyploids

**Figure 5:**
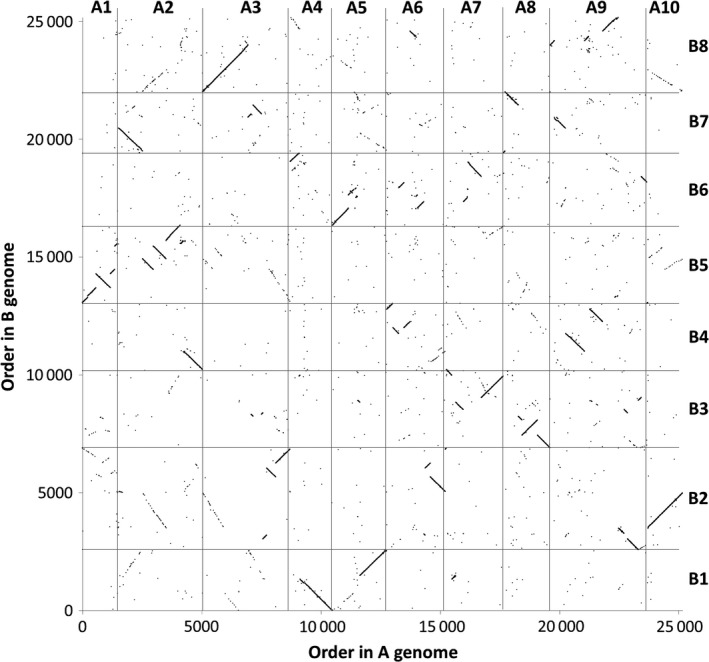
Homoeology relationships between *Brassica* A and B genomes. 25 167 pairs of homoeologous genes in the *Brassica* A and B genomes are plotted by their order in the respective genomes.

**Figure 6:**
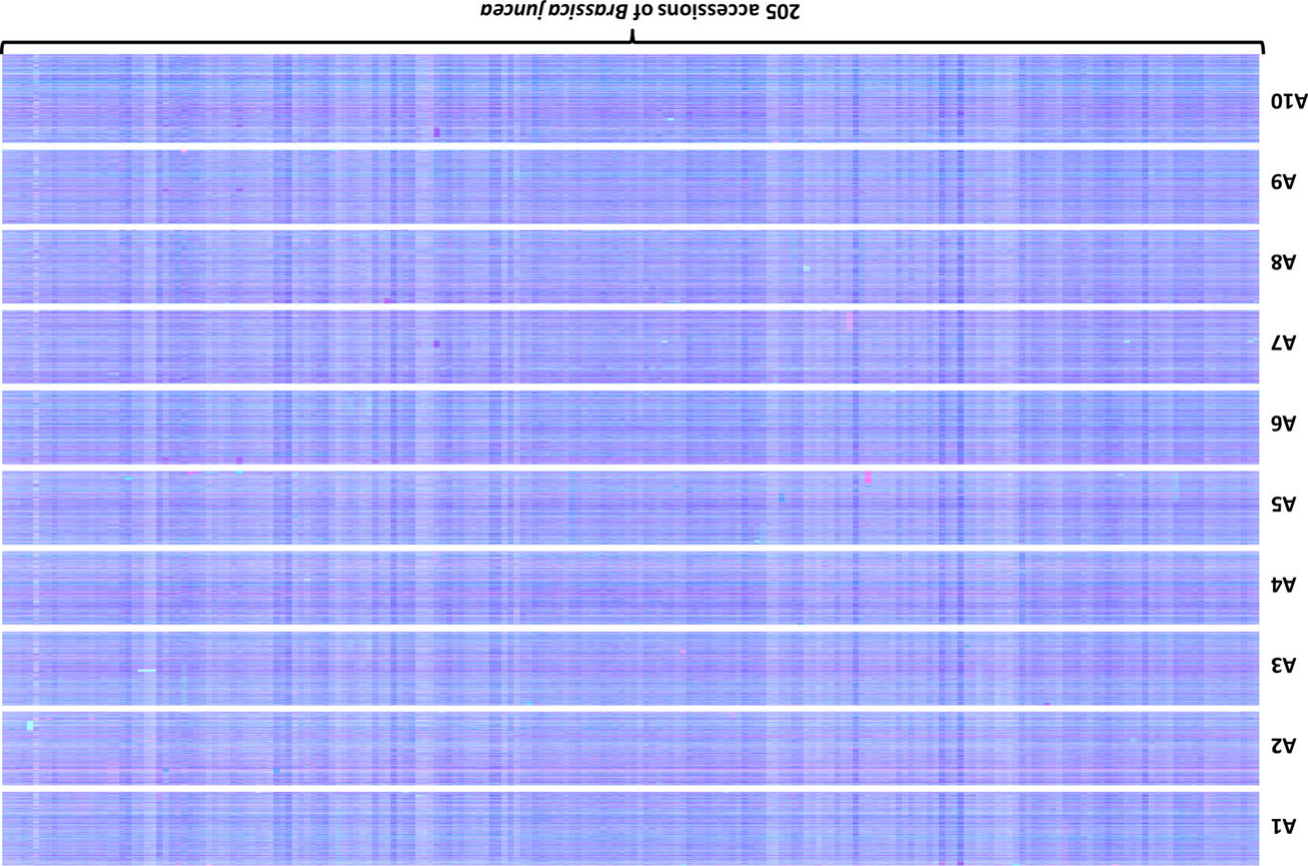
Visualization of homoeologous genome exchanges in *Brassica juncea* CGAT diversity panel using Transcriptome Display Tile Plots based on mRNAseq data. The relative transcript abundance of A and B genome homoeologous gene pairs is represented in CMYK colour space, with cyan component representing transcript abundance of the *Brassica* A genome copy and magenta component representing transcript abundance of the *Brassica* B genome copy. The pairs are plotted in *Brassica* A genome order (chromosomes denoted A1 to A10).

The distribution of HEs across the genome in the *B. napus* genetic diversity panel appears not to be random. Analysis of the frequency of occurrence of HEs across the panel (Data S7) confirms that the distribution of HEs is highly skewed towards certain regions of the genome. Alignment of the genome segments frequently involved in such HEs shows these relate to the set of instances where the corresponding regions in both genomes extend to telomeres. Alignment of genome regions rarely involved in such HEs shows these correspond to the set of instances where the regions of the A genome are (relative to the C genome) rearranged, with the sequences homoeologous to C genome telomeres being internal to A genome chromosomes. This suggests that HEs occur most frequently where homoeologues are able to pair over long regions extending to telomeres. To test this hypothesis we identified, a suitable polyploid species related to *B. napus* that contains genomes with less collinearity: *Brassica juncea* (an allotetraploid formed by hybridization of *B. rapa*, contributing the A genome and *Brassica nigra*, contributing the B genome), which is the principal oil crop in India (mustard rape). We identified, based on available *B. rapa* and *B. nigra* genome sequence resources, a set of 25 167 homoeologous gene pairs (Data S8), which define the homoeology relationships between the genomes, as shown in Figure 5, confirming that they exhibit less collinearity than those of *B. napus* (as shown in Figure 1). To assess the germplasm used by breeders, we produced TDTPs for the 205 accession ‘CGAT’ *B. juncea* genetic diversity panel (Data S9). The results are shown in Figure 6 and reveal a much lower incidence of HEs than had been observed in the *B. napus* germplasm and, again in contrast to *B. napus*, those that are present are mainly small segments internal to chromosomes. The lower rate of HEs extending to telomeres is consistent with the hypothesis that HEs occur most frequently where homoeologues are able to pair over long regions extending to telomeres.

**Figure 7:**
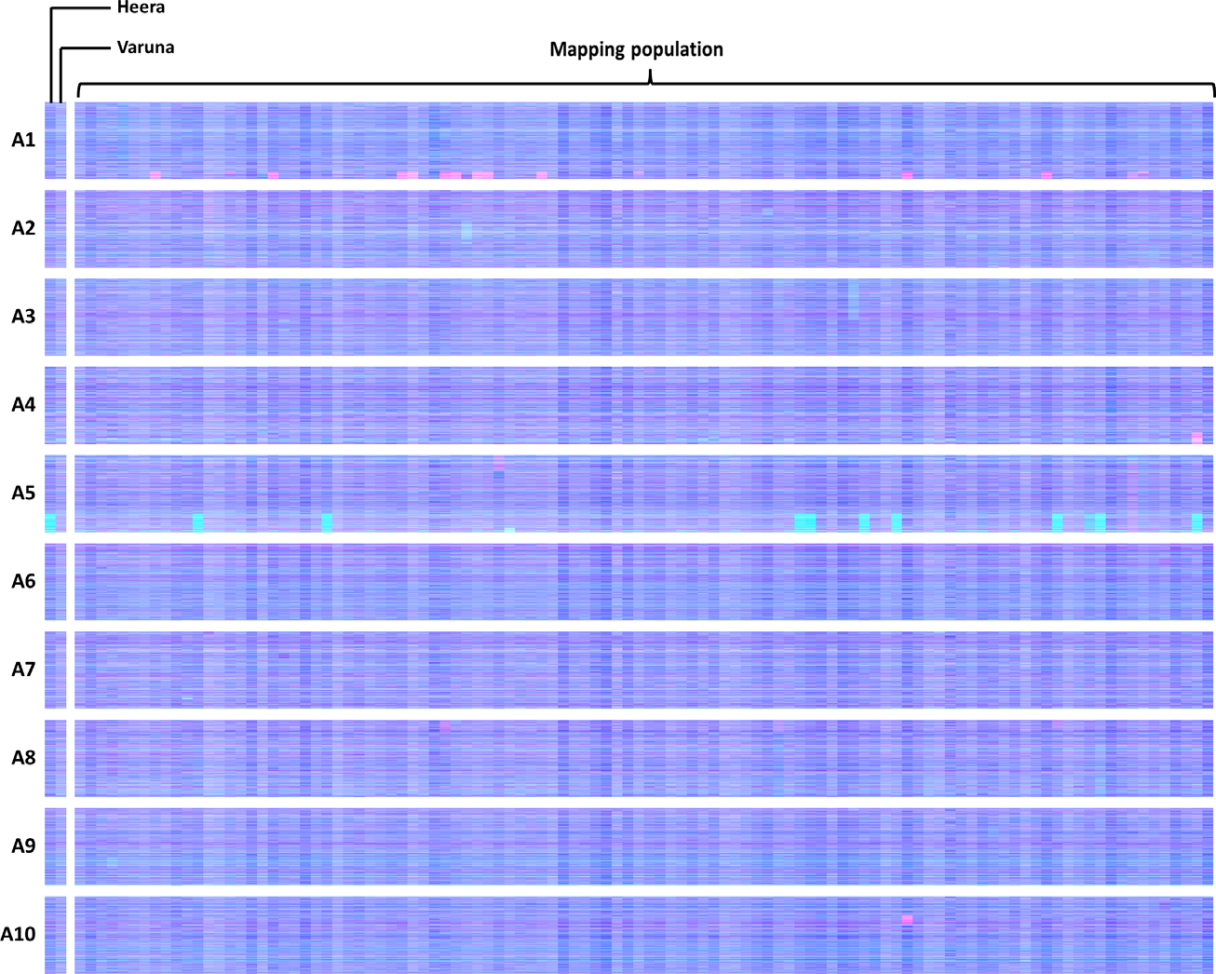
Visualization of homoeologous genome exchanges in VHDH population. The relative transcript abundance of A and B genome homoeologous gene pairs is represented in CMYK colour space, with cyan component representing transcript abundance of the *Brassica* A genome copy and magenta component representing transcript abundance of the *Brassica* B genome copy. The pairs are plotted in *Brassica* A genome order (chromosomes denoted A1 to A10).

The breeding of *Brassica* crops sometimes includes the production of doubled haploid (DH) plants (Möllers and Iqbal, 2009). The process involves colchicine treatment (to induce chromosome doubling) and results in completely homozygous plants. However, colchicine treatment inhibits meiotic telomere clustering (Cowan and Cande, 2002), so could affect the rate of HEs as collinearity extending to telomeres appears to be important. DH formation was used for the production of the *B. juncea* linkage mapping population VHDH (Pradhan *et al*., 2003). We therefore analysed this population for HEs to assess whether the process of DH production might increase the frequency of HEs occurring in this species despite the relatively lack of collinearity between its genomes. The results are shown in Figure 7 and reveal that numerous HEs are present in the population, in addition to clear segregation of an HE already represented in one of the parents, suggesting that DH production may indeed result in an elevated rate of HEs.

### The identification of homoeologous genome exchanges in bread wheat

**Figure 8:**
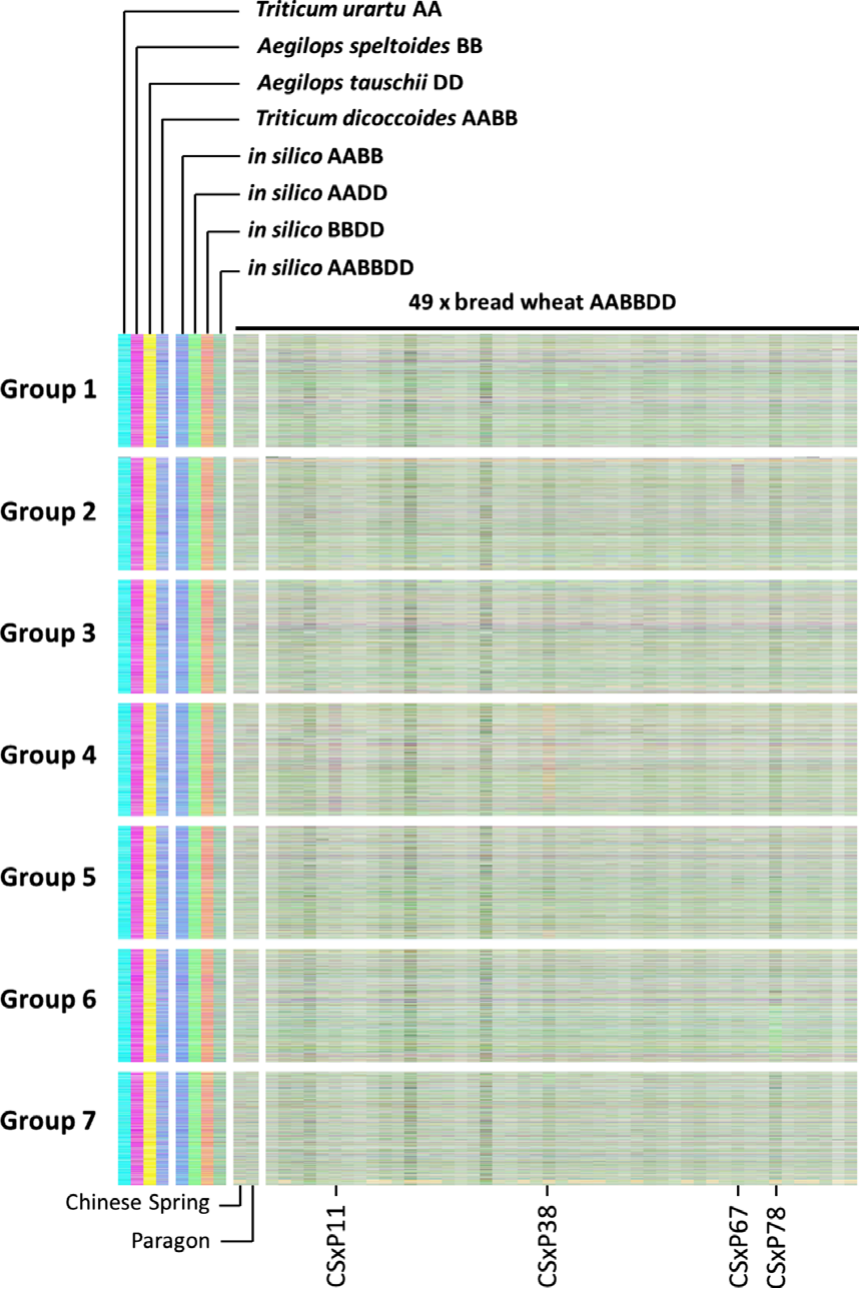
Visualization of homoeologous genome exchanges in wheat using Transcriptome Display Tile Plots based on mRNAseq data. The relative transcript abundance of A, B and D genome homoeologous gene triplets is represented in CMYK colour space, with cyan component representing transcript abundance of the wheat A genome copy, magenta component representing transcript abundance of the wheat B genome copy and yellow component representing transcript abundance of the wheat D genome copy. The triplets are plotted in genome order for the parents of a linkage mapping population (Chinese Spring × Paragon), 47 members of that population and controls comprising wheat parental species and *in silico* combinations to render resulting colours.

To assess the applicability of the approach to the identification of genome structural changes in further polyploid crops, we used it to analyse the genome of bread wheat. We identified homoeologous triplets (15 527 in total) and assigned transcript abundance to colour space using mRNAseq reads: A genome represented by cyan, B genome by magenta and D genome by yellow, as described previously for the analysis of potential genome dominance effects (Harper *et al*., 2016). We analysed a linkage mapping population derived by single seed decent (six generations) from a cross between cultivars Chinese Spring (CS) and Paragon. The results of the analysis of 47 lines, plus the parent cultivars, are shown in Figure 8. This analysis revealed segregation in the population of a small homoeologous exchange present in the parent line CS (near the bottom of group 7 chromosomes) and large, newly arising exchanges affecting groups 2 (CSxP67) and 6 (CSxP78) chromosomes. An additional copy of chromosome 4B was identified in CSxP11, and chromosome 4A has been lost from CSxP38. This analysis not only confirms the applicability of the method beyond *Brassica* species, but also shows that in this key polyploid crop, even in lines developed without treatment with colchicine, structural genome change occurs on the timescale of a few generations.

## Discussion

The repurposing of TDTPs for the visualization of homoeologous genome exchanges in allopolyploid crops makes tractable the analysis of germplasm collections used for breeding. Compared with the methods available previously, the mRNAseq‐based approach is rapid and inexpensive. There will be limits. For example, sequencing‐based approaches will not be able to discriminate between the genomes of autopolyploid species, although quantitative analysis of transcript abundance may still be indicative of dosage (copy number) variation. Strong genome dominance effects, for example where one genome of an allopolyploid is transcriptionally silenced, would permit observation of doubling of the expressed genome as intensified signals, but copy number of the silenced genome could not be determined. A great advantage, however, is that genomics resources enabling the hypothetical ordering of genes are necessary only for one of the genomes being analysed to generate the TDTPs.

mRNAseq‐based detection of genome structural variation arising, in polyploid species, from homoeologous exchange, has led to the recognition that such events are very frequent and segregating widely in the germplasm used by breeders of *B. napus* crops. The distribution of regions of the genome in which exchanges have occurred is not random, but predominantly in those showing greatest collinearity extending to telomeres. This is consistent with the hypothesis that HEs will occur where homoeologues are able to pair most efficiently and the observation that HEs mostly appear to extend to telomeres in both genomes (albeit often with alternating exchanges indicative of subsequent recombination events) is consistent with the hypothesis that subtelomeric regions are involved in partner recognition and selection (Corredor *et al*., 2007). It is notable also that HEs detected are conservative; that is, they involve substitution of genome segments. This may reflect selection imposed during breeding of individuals in which HEs have been nonconservative, *that is* where some of the exchanged region between the point of recombination and the telomere is nonhomoeologous, are impaired in growth or productivity. Although several of the regions frequently involved in HEs in *B. napus* show no bias, the majority mainly involves substitution of C genome sequences by A genome sequences, giving rise to the visual skewing towards cyan in the genetic diversity panel, as shown in Figure 2. Although the reasons for this bias are unclear, one possibility is that interspecific crosses have been conducted between *B. napus* and *B. rapa*, resulting in an increase in the genetic diversity of the A genome, but some of the HEs may have involved substitution of *B. napus* C genome by *B. rapa*‐derived A genome sequences.

**Figure 9:**
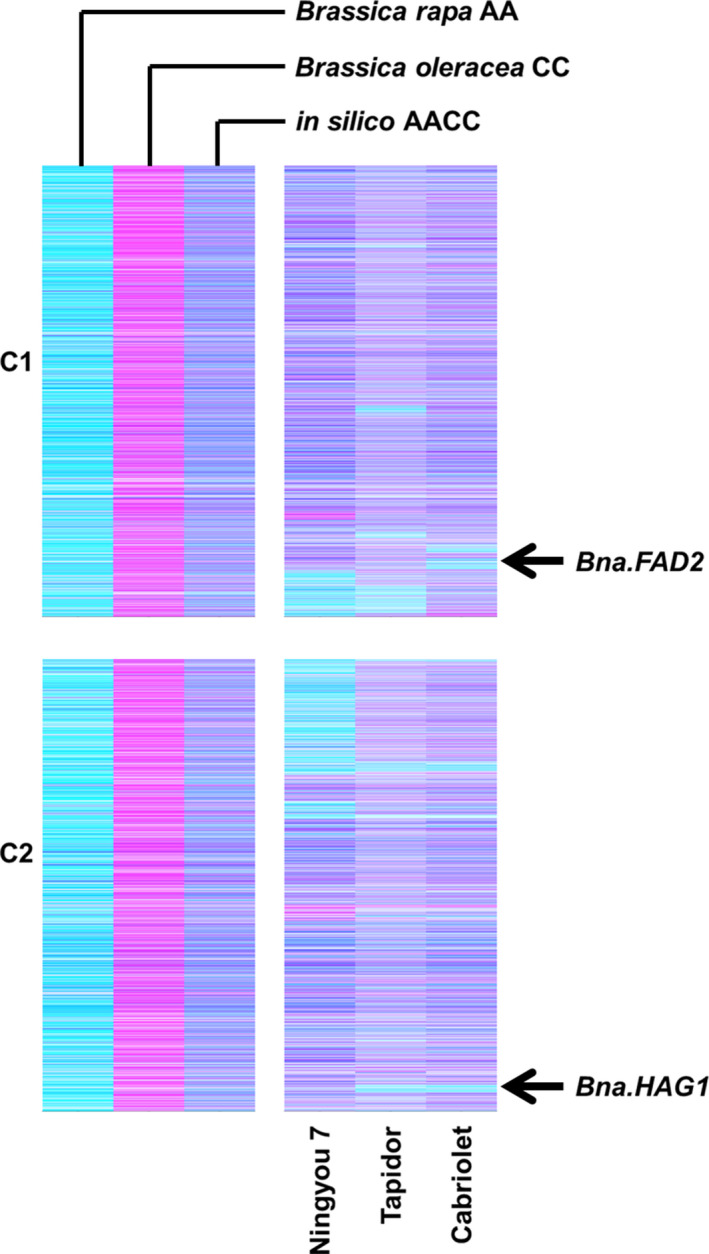
Visualization of homoeologous genome exchanges in *Brassica napus* causative of trait variation. The relative transcript abundance of A and C genome homoeologous gene pairs is represented in CMYK colour space, with cyan component representing transcript abundance of the *Brassica* A genome copy and magenta component representing transcript abundance of the *Brassica* C genome copy. The pairs are plotted in *Brassica* C genome order (only C1 and C2 shown) along with controls comprising parental species and their *in silico* combination. The positions of *B. napus* genes implicated in control of trait variation are marked.

Genome visualization by TDTPs will have a broad impact on crop improvement due to the effects that HEs can have on traits of importance. Two examples are illustrated in Figure 9. The first example is the HE that substitutes a region near the bottom of chromosome C2 with homoeologous A genome sequences. This exchange removes from varieties Cabriolet and Tapidor a functional orthologue of *HAG1*, which controls seed glucosinolate content, an important quality trait in rapeseed. The second example is the HE that substitutes a region towards the bottom of C1 with homoeologous A genome sequences. This exchange removes from variety Cabriolet a functional orthologue of *FAD2*, which controls the amount of polyunsaturated fatty acids synthesized and correspondingly the oleic acid content of seed oil, another important quality trait in rapeseed. Both events had originally been described as apparent deletions from the C genome as the increased copy number of the corresponding A genome sequences could not be identified by the analysis undertaken in either case (Harper *et al*., 2012; Wells *et al*., 2014). The ability to detect cost‐effectively homoeologous genome exchanges across large panels of plants permits the systematic association of phenotypic variation with genome structural change, particularly the identification and selection of genome deletions or duplications encompassing genes with trait‐controlling dosage effects.

## Experimental procedures

### Identification of homoeologous gene pairs and triplets

To assess genome exchange patterns in *Brassica* species, homoeologous gene pairs were identified via a two‐way reciprocal BLASTn analysis (threshold E‐value 1E‐30). This analysis used the *Brassica* pan‐transcriptome CDS models for *Brassica* A and C genomes (He *et al*., 2015) and *Brassica* B genome CDS models from *B. nigra* (I.A.P Parkin, unpublished). About 32 904 gene pairs were identified for *B. napus* (A and C genomes) and 25 167 gene pairs were identified for *B. juncea* (A and B genomes). For wheat, 15 527 homoeologous gene triplets had been identified previously (Harper *et al*., 2016).

### Growth and sampling of plants

Plants were sown on Levington professional F2 compost and grown in long day (16/8 h, 20 °C/14 °C) glasshouse conditions. Second true leaves from each of four plant replicates per accession were harvested when they reached ~3 cm in diameter, as close to the mid‐point of the light period as possible. Leaves were harvested separately for processing as individual replicates or pooled when processed as a single sample per accession and immediately frozen in liquid nitrogen. Frozen leaf samples were stored at −80 °C.

### RNA preparation

Pooled frozen leaf samples were ground in liquid nitrogen. RNA was extracted using the manufacturer's instruction for Omega Biotek EZNA Plant RNA Kit.

### DNA preparation

DNA was extracted from individual replicate samples. The samples were homogenized in lysis buffer with 3‐mm metal beads using Qiagen (Manchester, UK) TissueLyser II (30/s, 2 min). BioSprint 96 DNA Plant Kit and Qiagen BioSprint 96 Workstation system were used for the DNA extraction.

### Transcriptome sequencing

Illumina sequencing, quality checking and processing were conducted as described previously (Higgins *et al*., 2012) except that 100 base reads obtained from the HiSeq2500 platform were used. Maq was used for mapping with default parameters, meaning that reads with no more than two mismatches with summed Q ≥ 70 were mapped.

### Transcript quantification

Using methods and scripts described in Bancroft *et al*. (2011) and Higgins *et al*. (2012), expression of each of 116 098 genome‐mapped *Brassica* CDS gene models was estimated for each of the accessions, using the recently developed ordered *Brassica* A and C pan‐transcriptomes (He *et al*., 2015) as reference sequences for *B*. *napus* panels of accessions. Expression of each of 87 630 genome‐mapped *Brassica* CDS gene models representing the ordered *Brassica* A and B transcriptomes was estimated for each of the *B. juncea* accessions. Expression of each of 147 411 genome‐mapped wheat unigenes was estimated for each of the wheat accessions, as described previously (Harper *et al*., 2016). Transcript abundance was quantified and normalized as reads per kb per million aligned reads (RPKM) for each sample.

### Analysis of differential gene expression

Differential gene expression was analysed in leaves for 27 accessions of *B. napus*, using four biological replicates (i.e. in leaves from four separate plants of each accession). Plants were grown, RNA extracted and purified, Illumina mRNA‐seq data produced and transcript abundance quantified as described above, and genes showing low transcript abundance (mean RPKM across the respective panel <0.4) were removed. For each pair of homoeologous CDS models, a *t*‐test analysis was performed between the expression profiles of a single accession (with four replicated samples) for one genome (A or C) against the mean of all samples of the 27 *B. napus* variety panel. The expression profiles of each homoeologous CDS model can be allocated into one of nine categories of genome dosage changes (Table 1). Differentially expressed CDS models are plotted with the same colour coding as the TDTP display. Homoeologue pairs showing significant (*P* < 0.01) differential expression were then placed in genome order for over‐expression relative to the mean of all samples (Data S3), under‐expression relative to the mean of all samples (Data S4) and over‐expressed with the other genome under‐expressed relative to the mean of all samples (Data S5).

### Transcriptome display tile plots

A visualization approach involving in‐house R script to display the relative transcript abundance of homoeologous gene pairs and triplets on a genome scale was used, as described previously (Harper *et al*., 2016). Briefly, each member of the homoeologous pair/triplet has a value (normalized from 1 to 0 for the population so that darker colouring shows higher transcript abundance and lighter colouring shows lower transcript abundance) assigned in CMYK colour space where the contributions from each genome copy are coded to cyan, magenta or yellow channels, and the results displayed using tile plots. For *B. napus*, A genome copies are assigned to cyan and C genome copies are assigned to magenta, resulting in predominantly blue (cyan/magenta combination) where both genes are represented in an accession. For *B. juncea*, A genome copies are assigned to cyan and B genome copies are assigned to magenta, resulting in predominantly blue (cyan/magenta combination) where both genes are represented in an accession. For wheat, A genome copies are assigned to cyan, B genome copies are assigned to magenta, and D genome copies are assigned to yellow, resulting in predominantly grey (cyan/magenta/yellow) where all three genes are represented in a line.

### Validation of homoeologous exchanges by low‐pass genome resequencing

To differentiate clearly between structural genome changes and long‐range gene silencing as an alternative explanation for apparent copy number reduction, we undertook low‐pass genome sequencing on one individual of each of the same 27 varieties of *B. napus* studied by transcriptome analysis. Illumina sequencing, quality checking and processing were conducted as described previously (Higgins *et al*., 2012) except that 100 base reads obtained from the HiSeq platform were used. BWA (Li and Durbin, 2009) sequence‐alignment program (v0.7.12) was used for mapping with default parameters. The sequence reference being used shares the same gene id as the transcript quantification for direct comparison, but spliced introns are included for better mapping. The redundancy of representation of gene models was quantified and normalized as reads per kb per million aligned reads (RPKM). To visualize regions of the genome involved in homoeologous exchanges in a comparable way to the mRNAseq‐based analysis, we used TDTPs to visualize the genome redundancy data, essentially as for visualization based on mRNAseq data. This involved assigning quantitative representation (as RPKM) for each member of the 19 083 A and C genome homoeologous gene pairs with representation >0.01 RPKM a value in CMYK colour space where the contributions from the A and C genome copies were coded to cyan and magenta channels, respectively, and displaying the results using tile plots.

## Supporting information


**Data S1** Illumina read mapping statistics.

**Data S2** Reciprocal BLAST Brassica AC genomes.

**Data S3** Conservative expressing Brassica AB genes.

**Data S4** Over‐expressing Brassica AB genes.

**Data S5** Under‐expressing Brassica AB genes.

**Data S6** *Brassica napus* diversity panel list.

**Data S7** Analysis of Brassica AC HEs.

**Data S8** Reciprocal BLAST Brassica AB genomes.

**Data S9** *Brassica juncea* diversity panel list.
