# Supplementary figures and images for: Extensive homoeologous genome exchanges in allopolyploid crops revealed by mRNAseq‐based visualization
Source: Plant Biotechnol J. 2016 Dec 6;15(5):594–604. doi: 10.1111/pbi.12657 (PMC5399007; doi:10.1111/pbi.12657)

C01

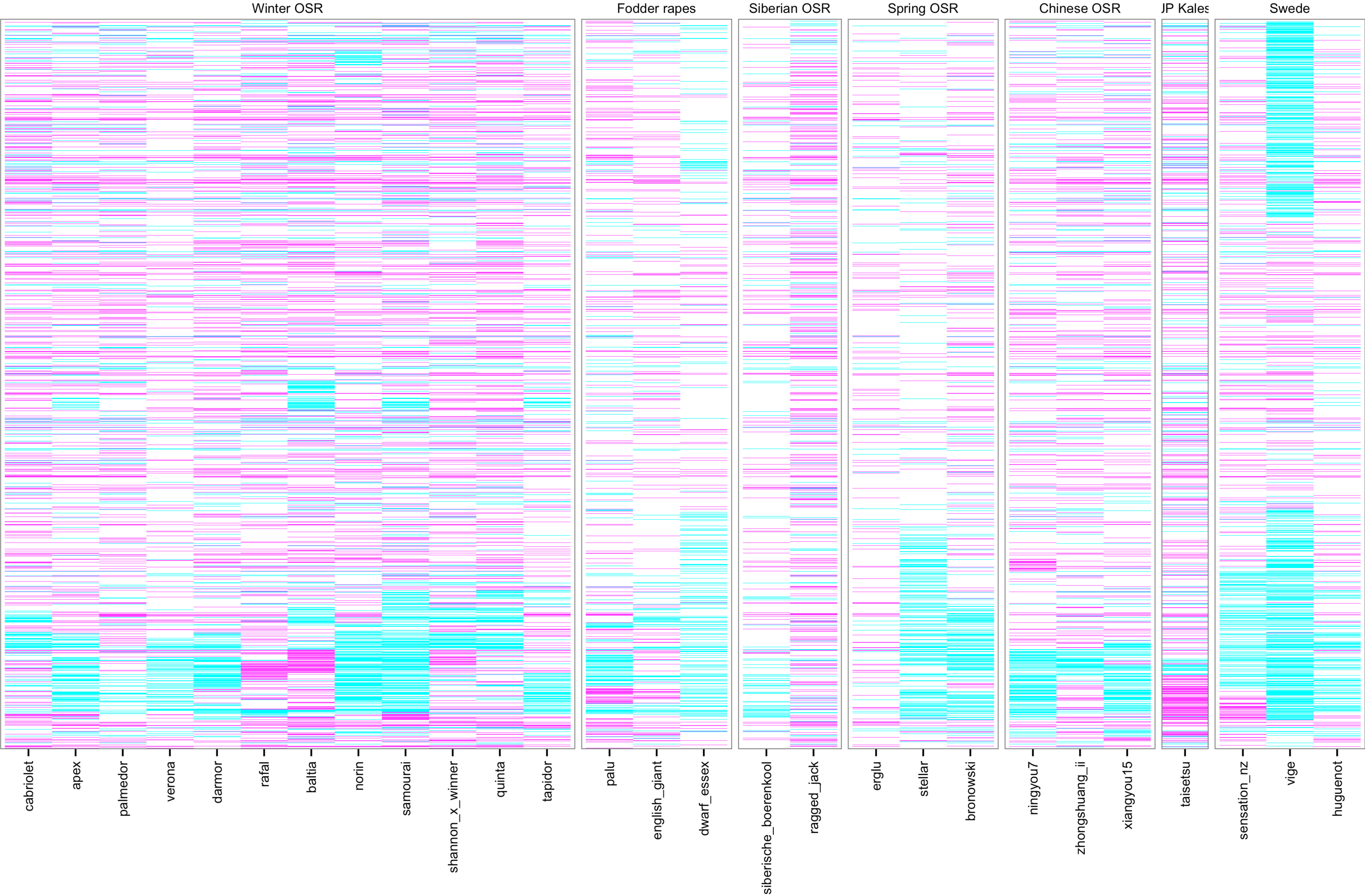

C02

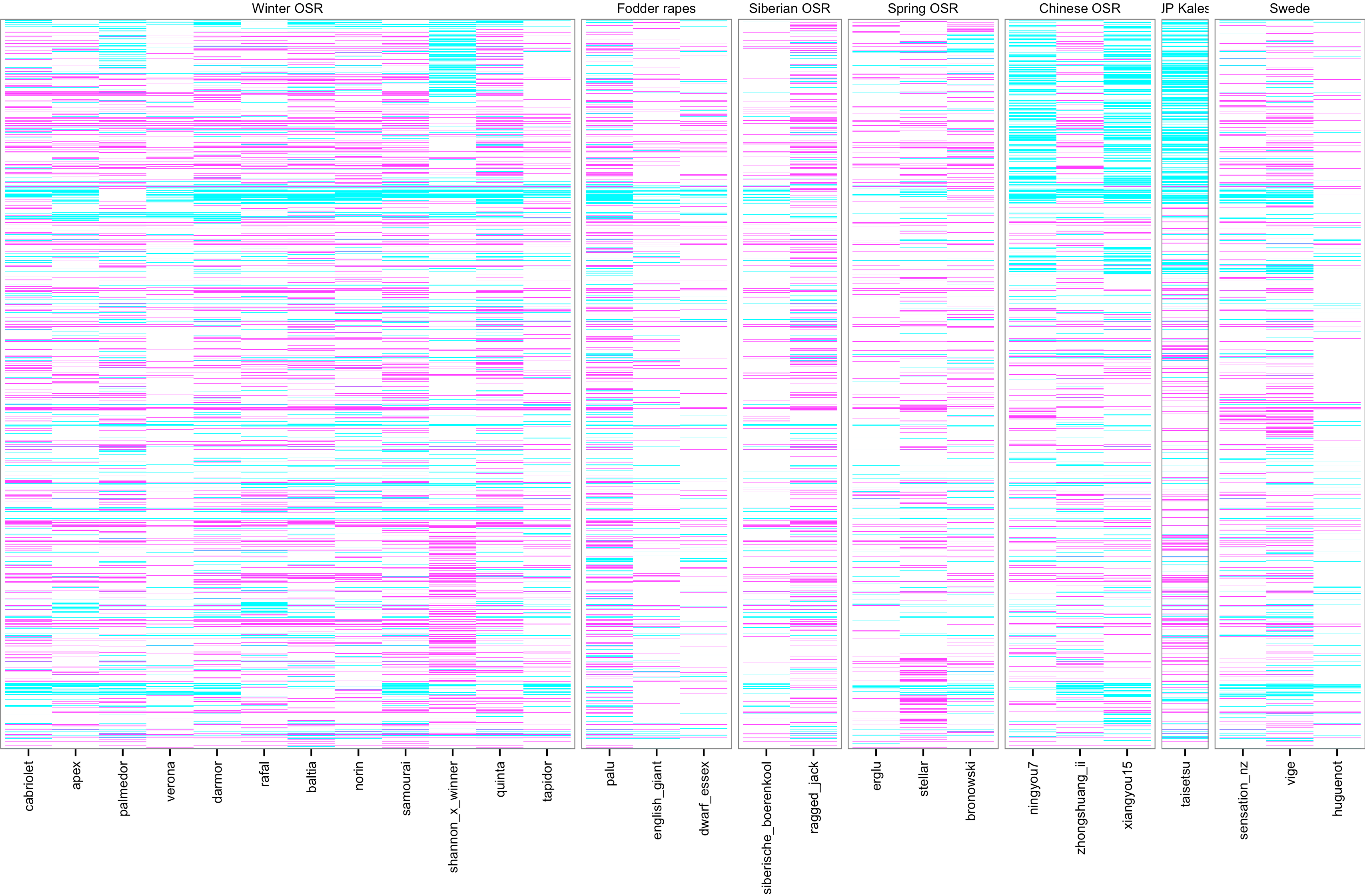

C03

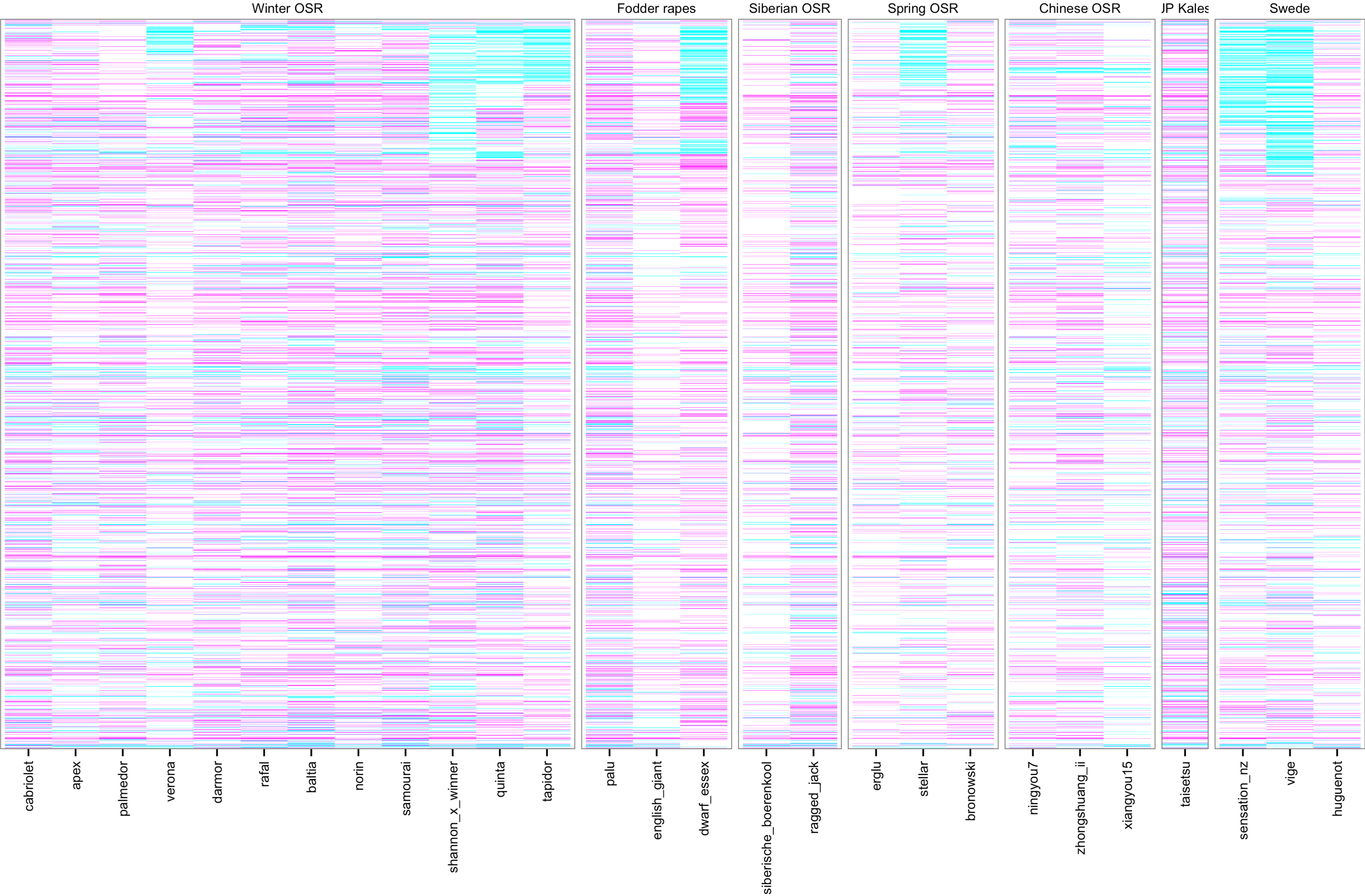

C04

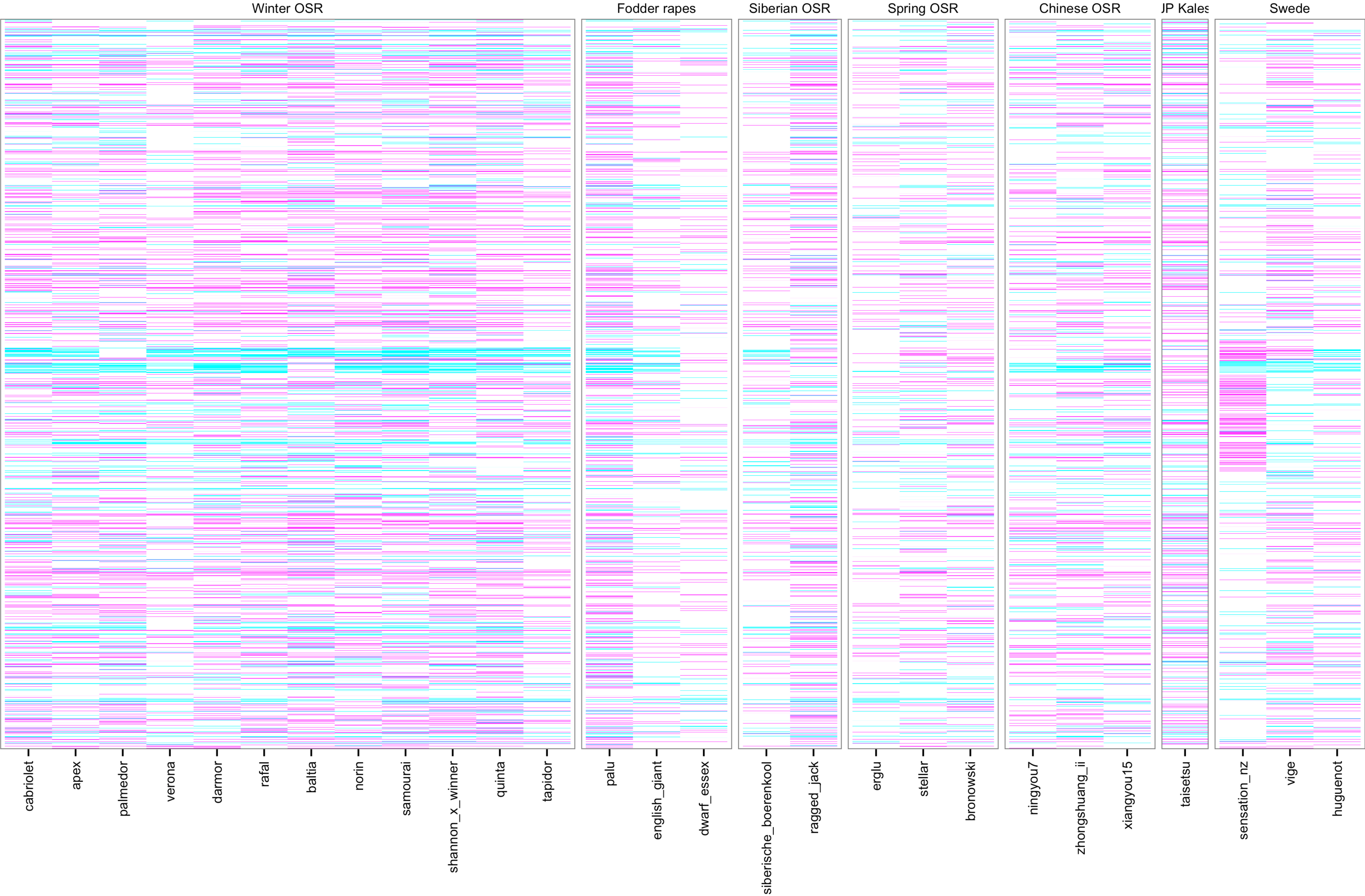

C05

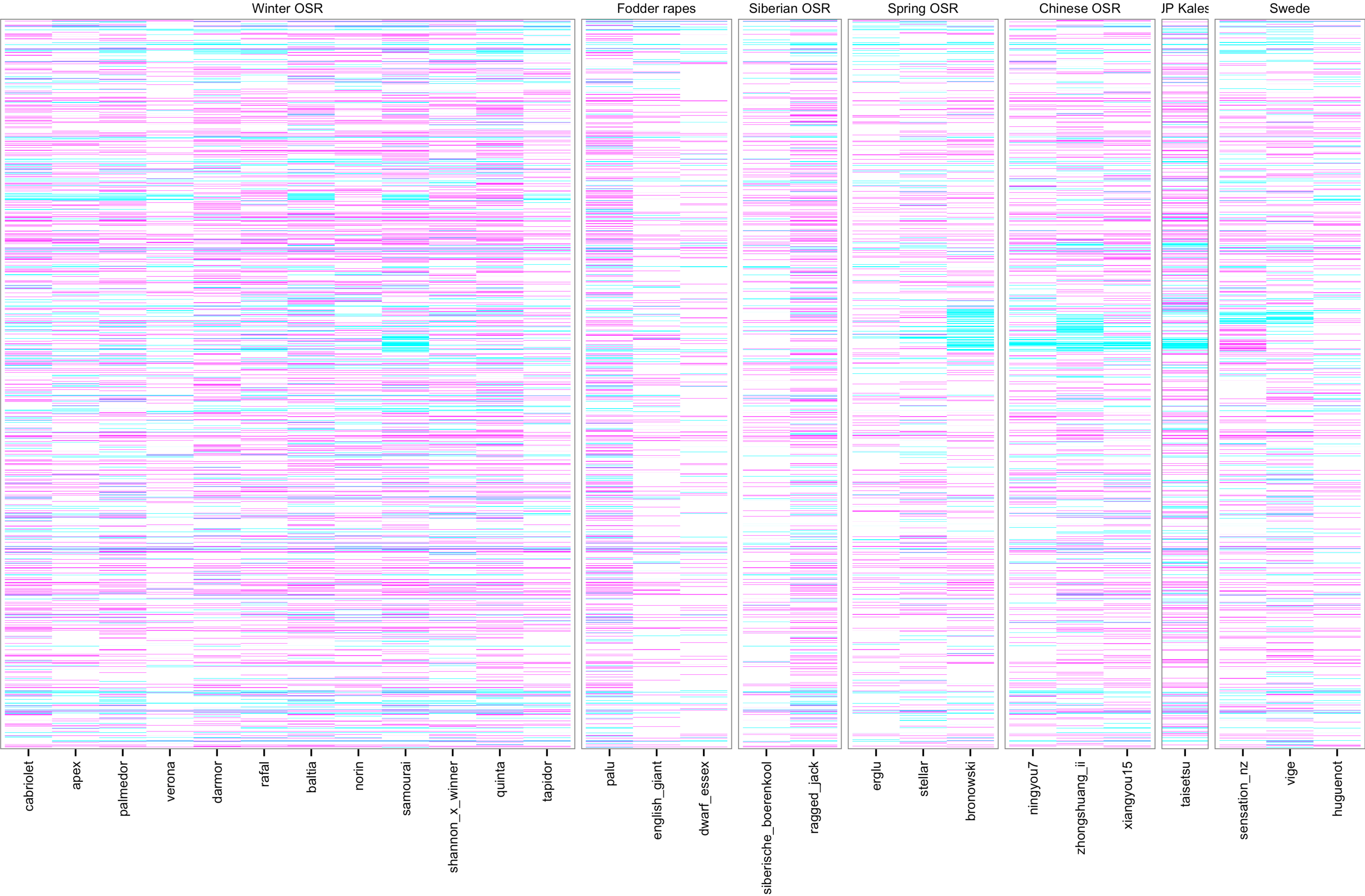

C06

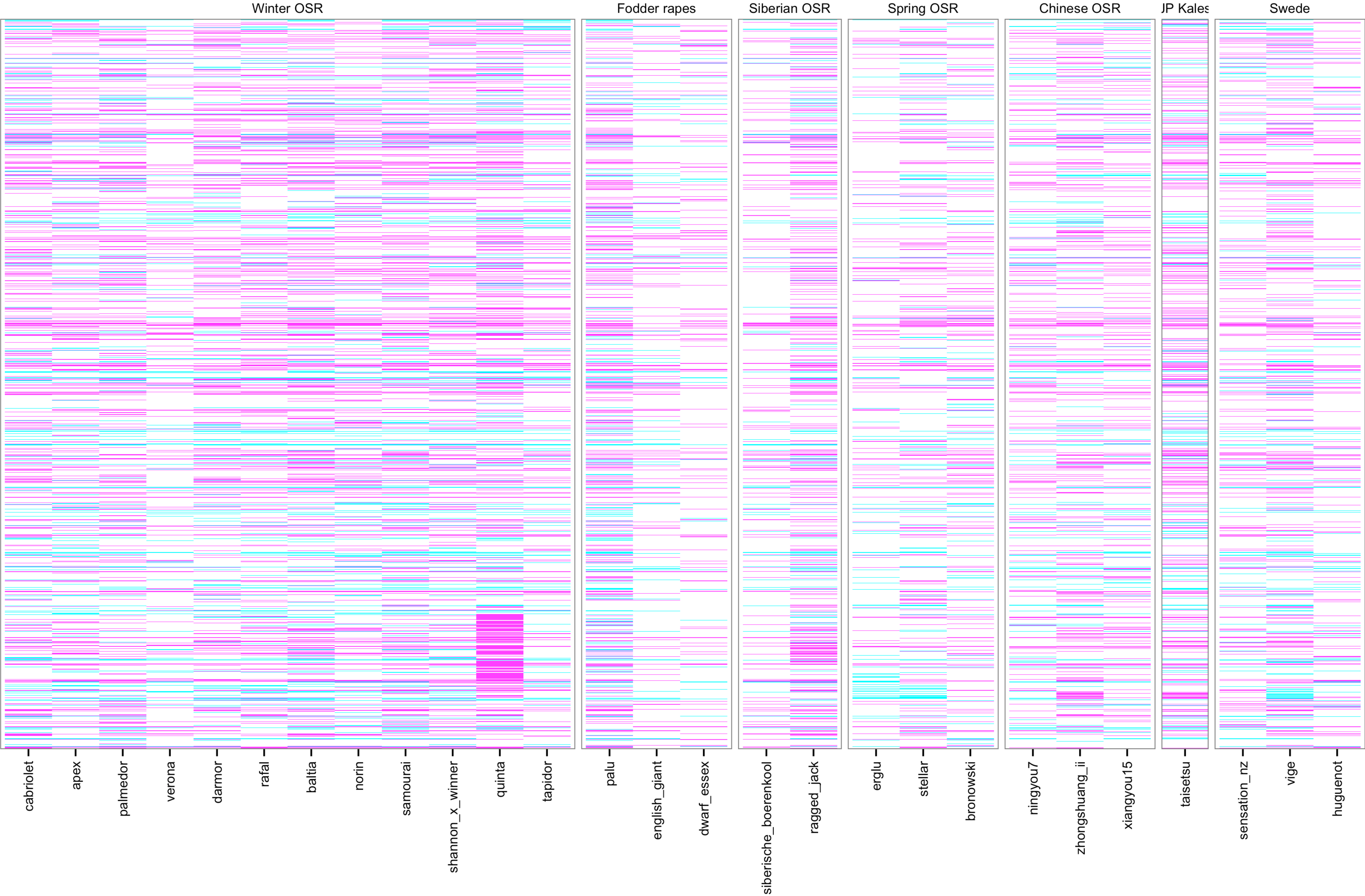

C07

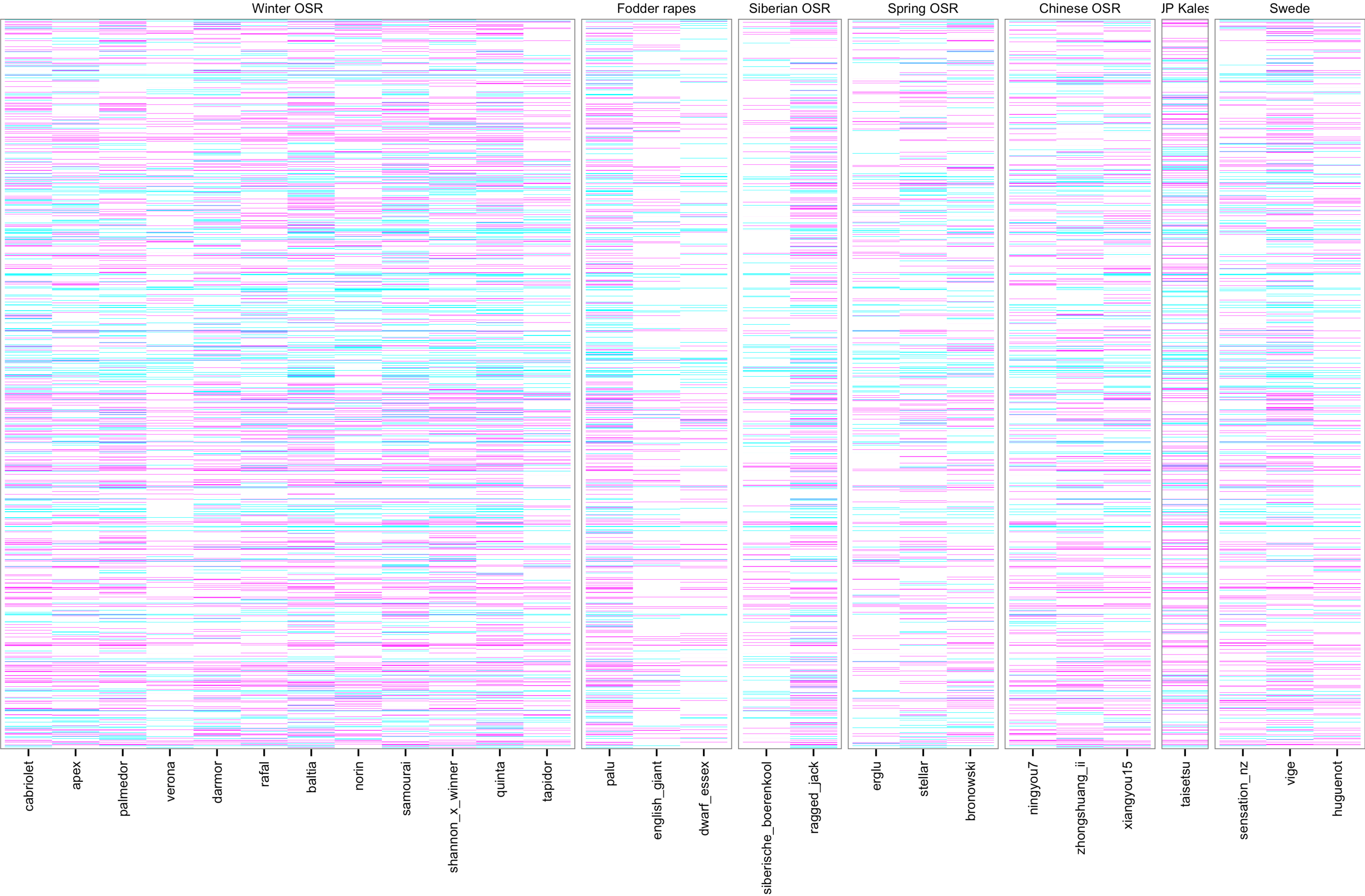

C08

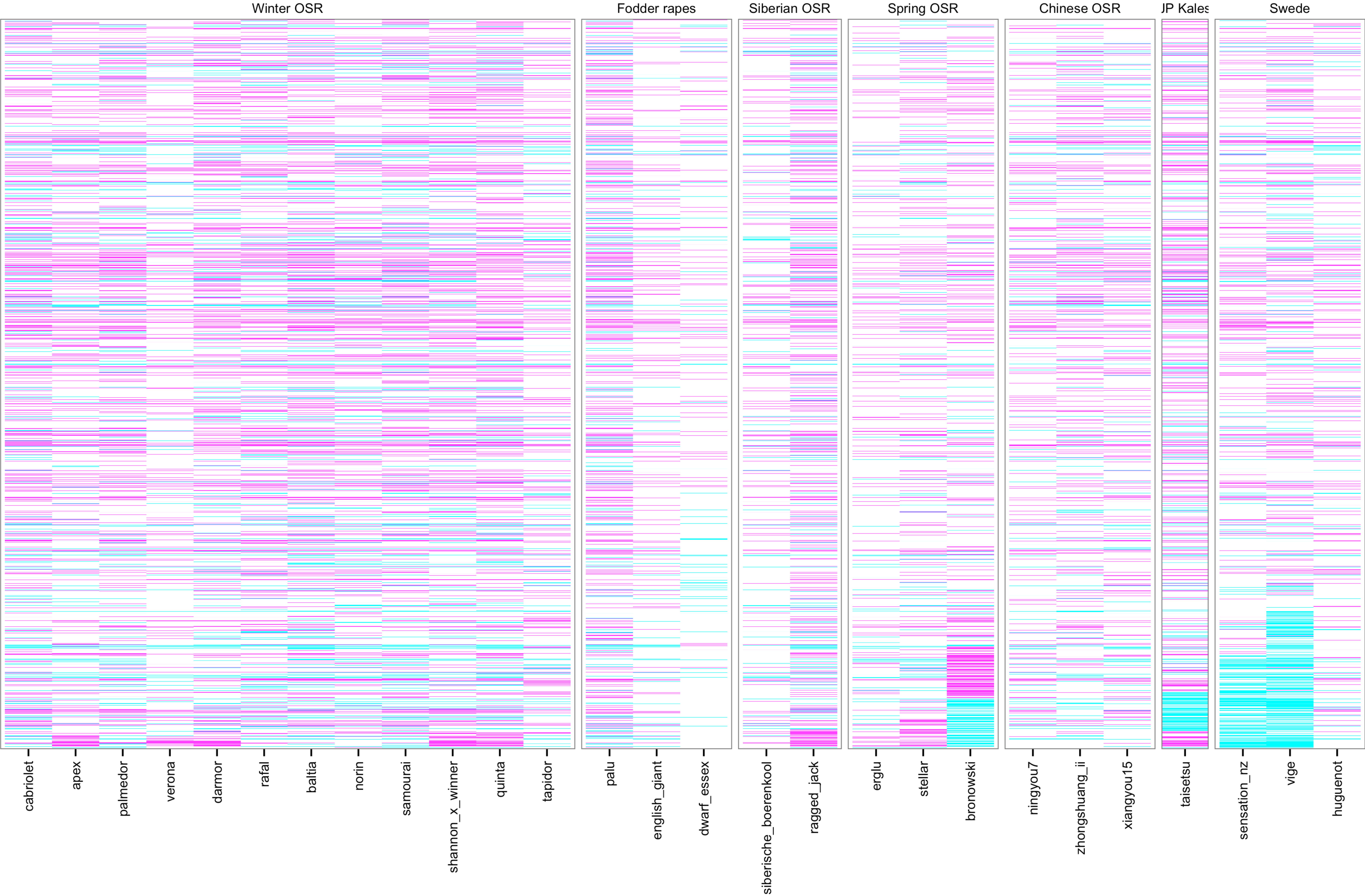

C09

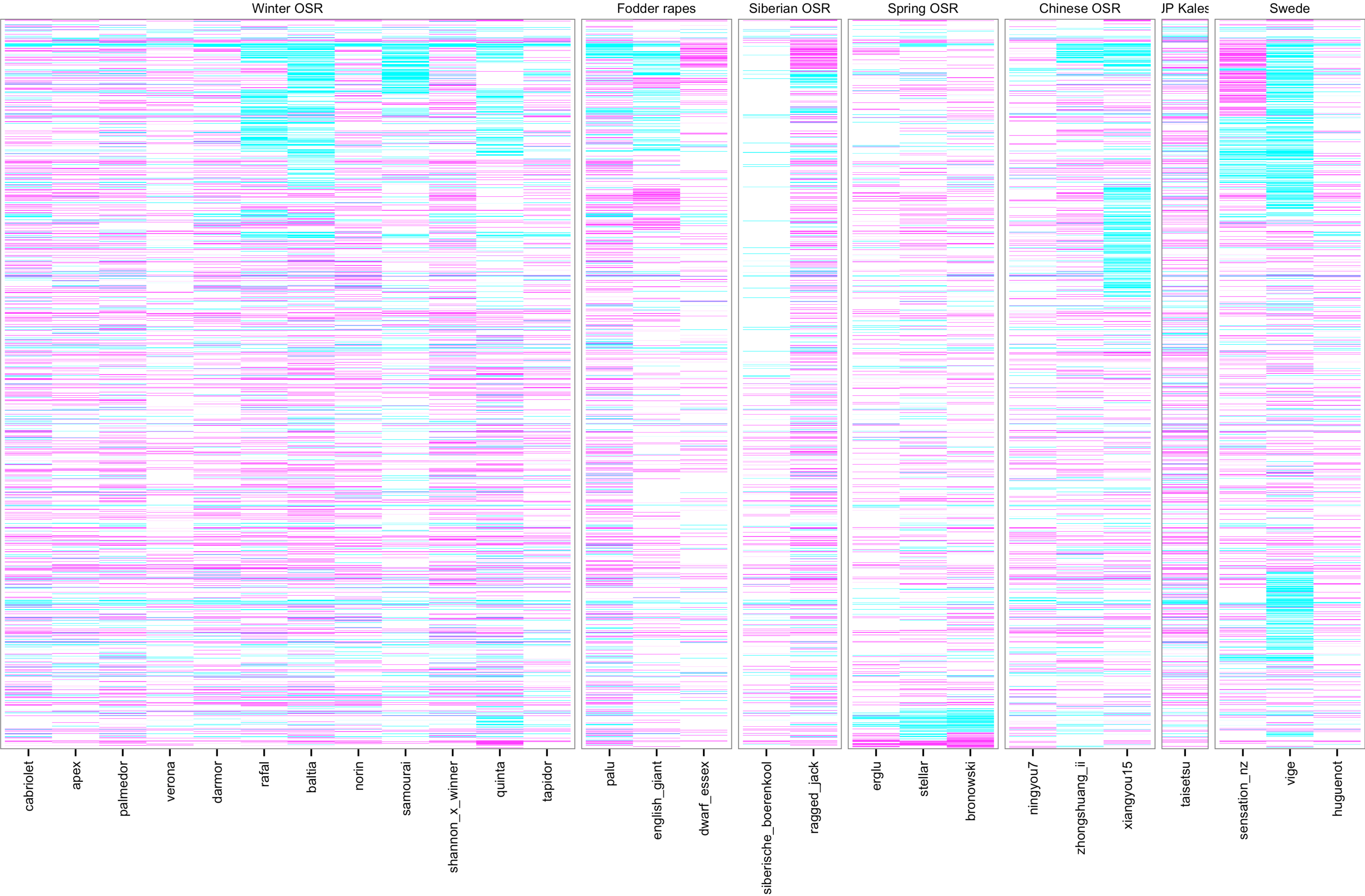

Supplement: Supplementary file 3 — Data S3 Conservative expressing Brassica AB genes. [file PBI-15-594-s006.pdf]

C01

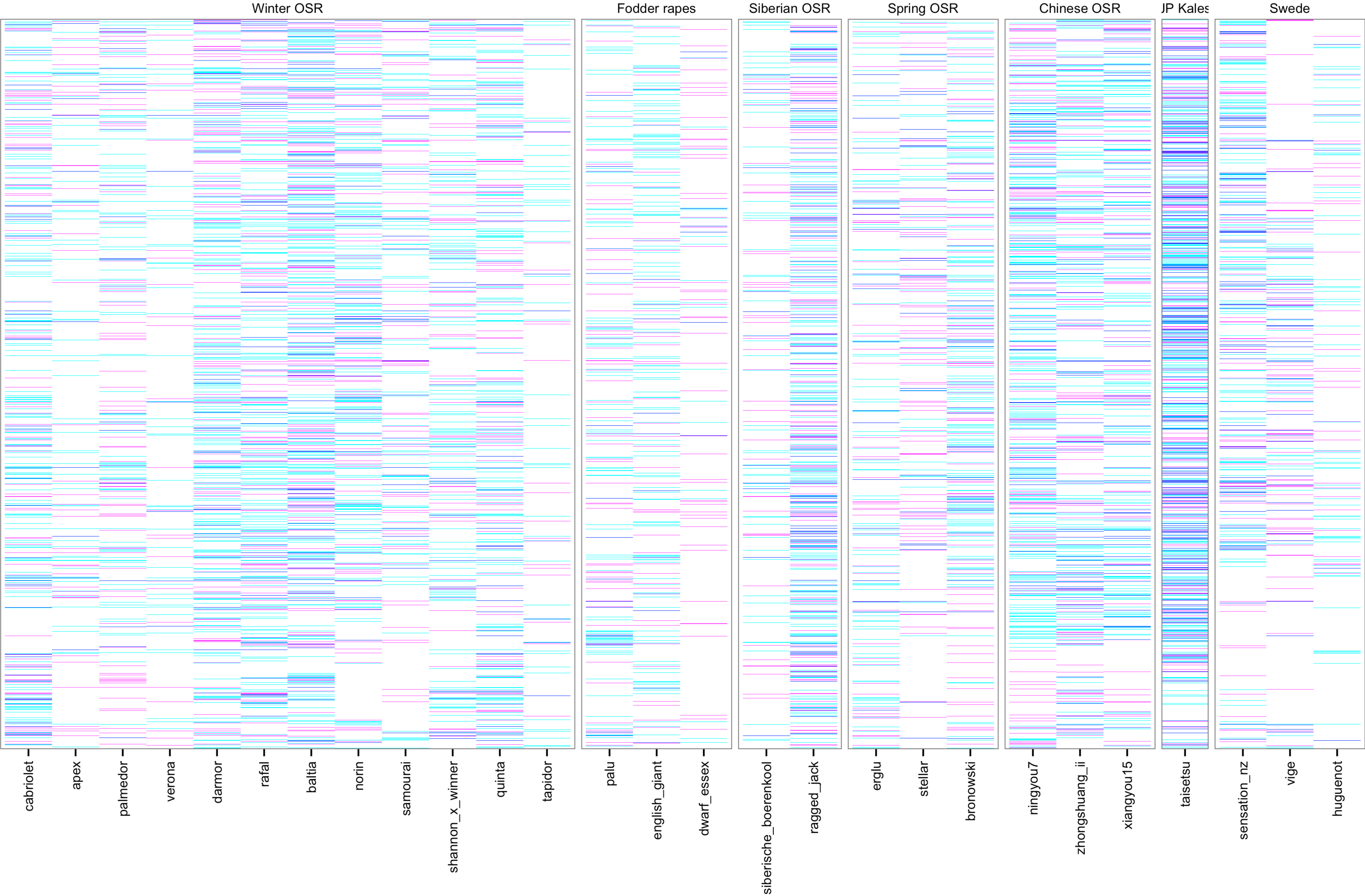

C02

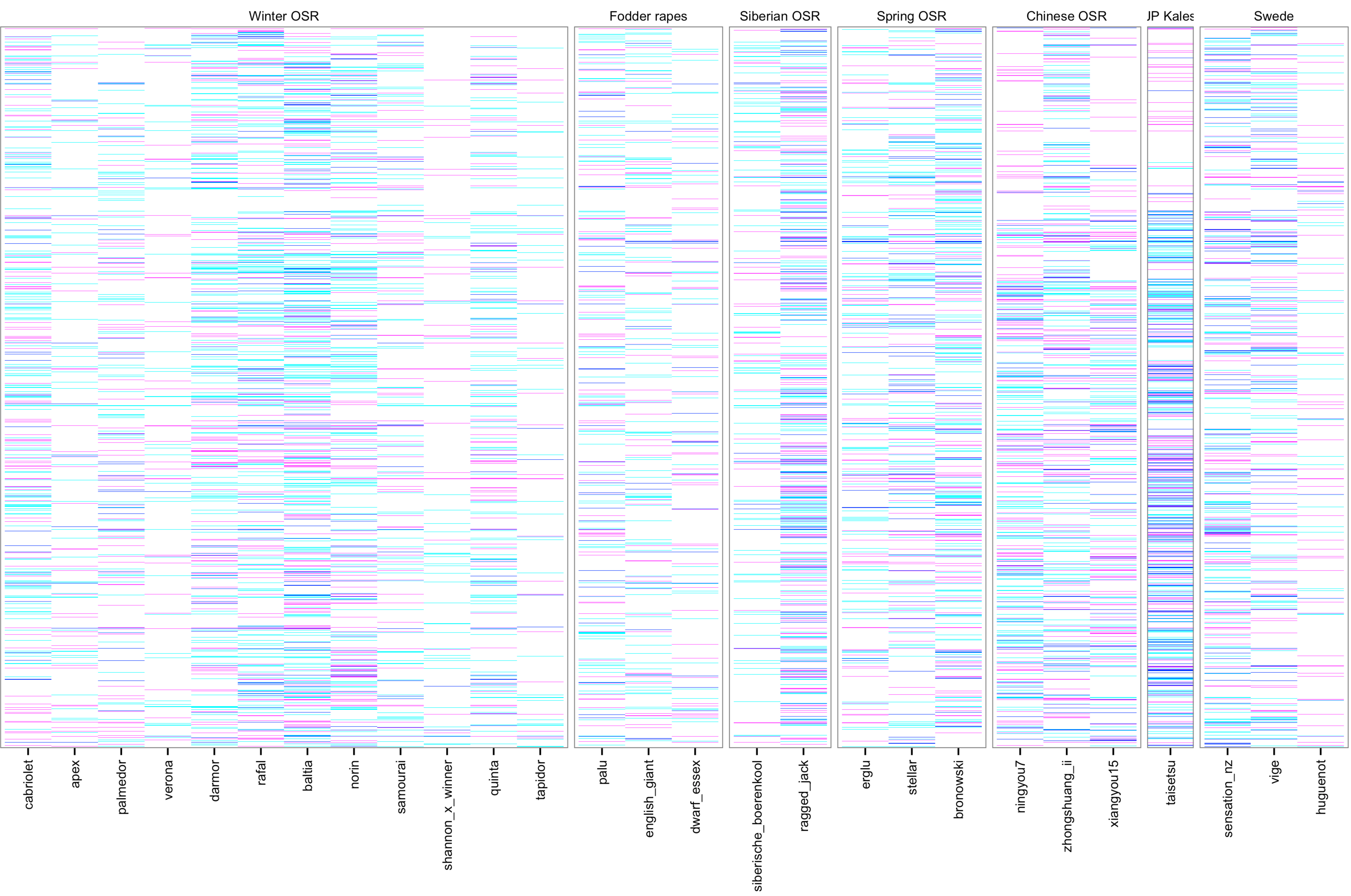

C03

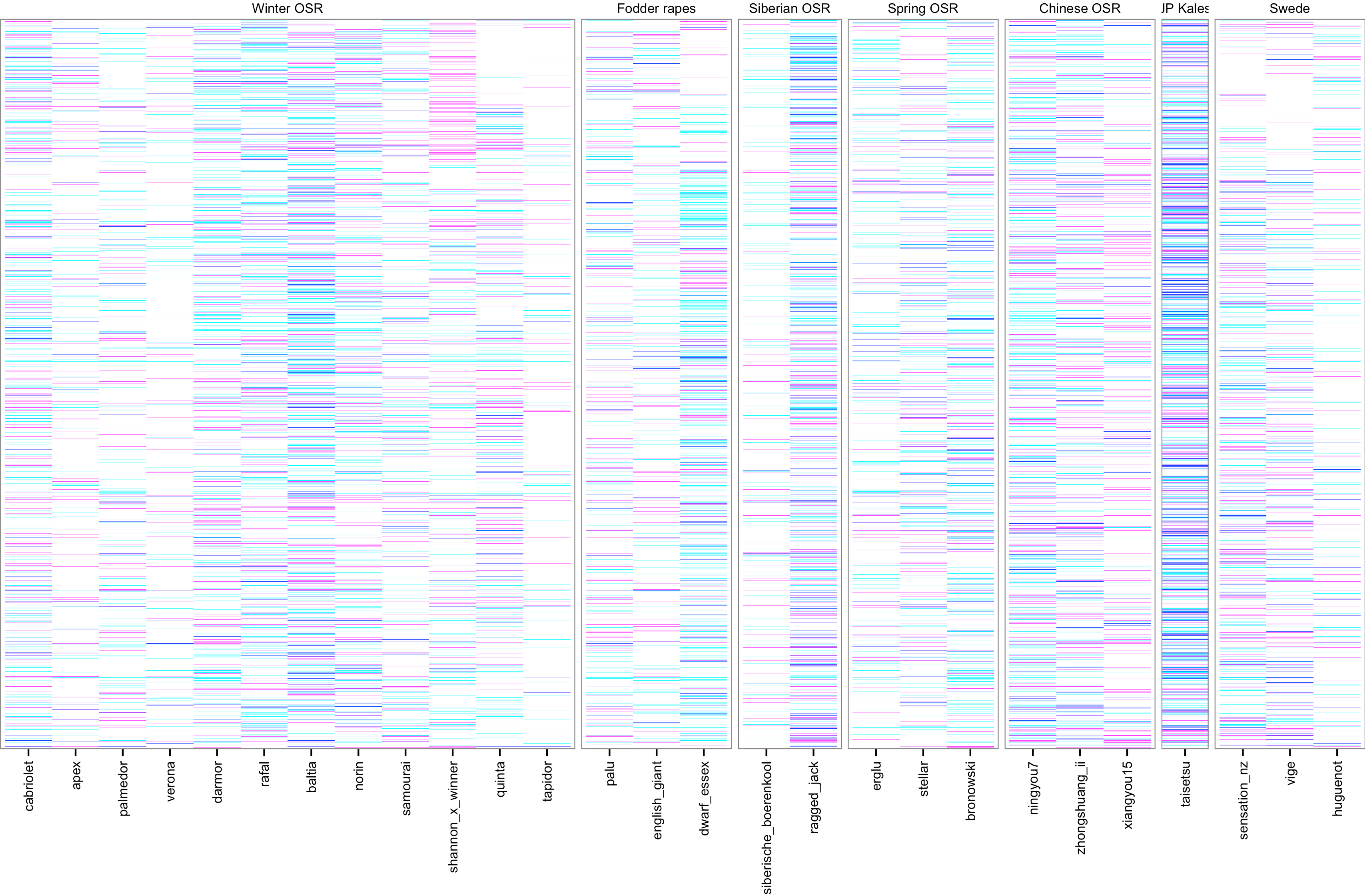

C04

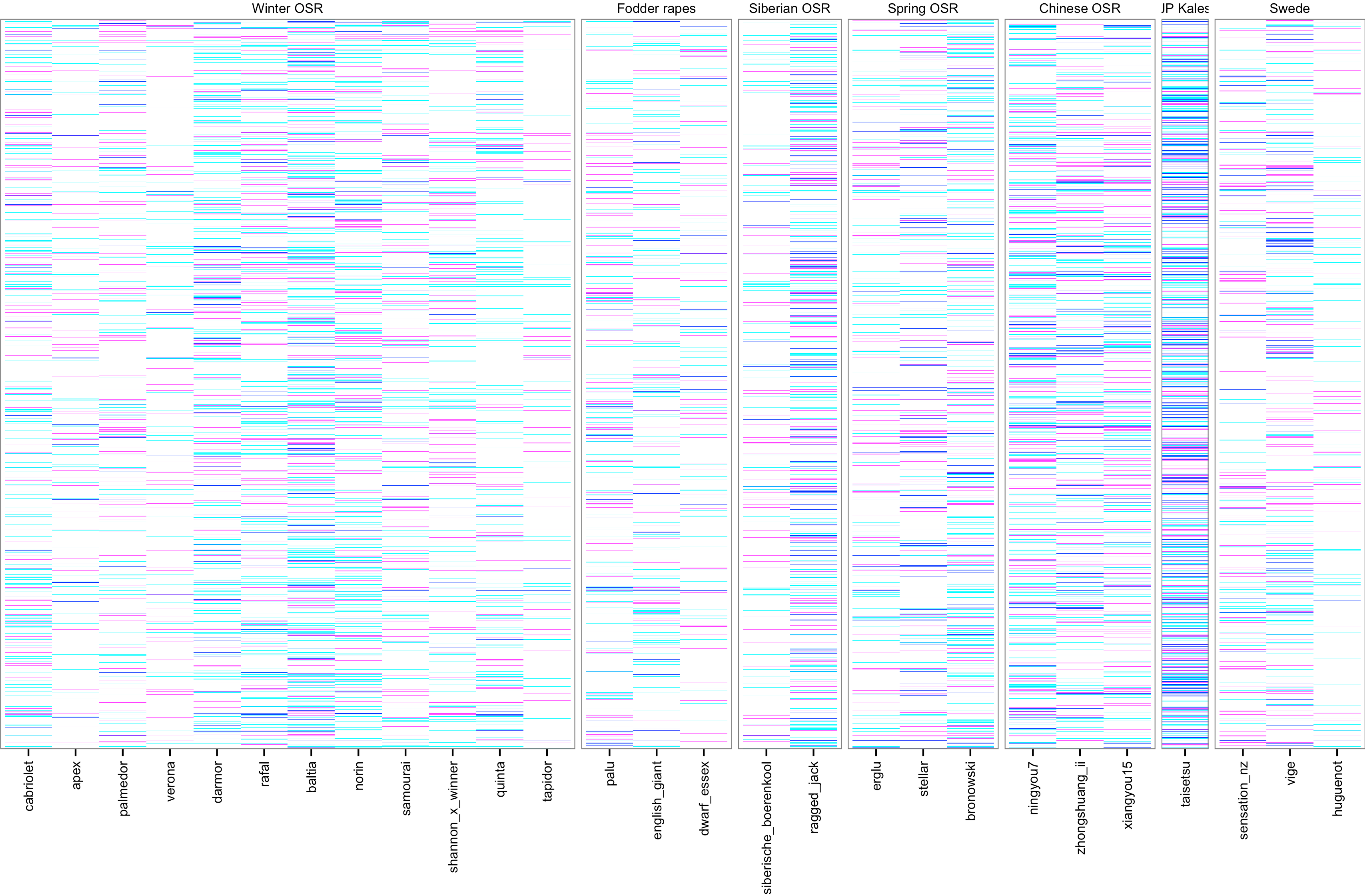

C05

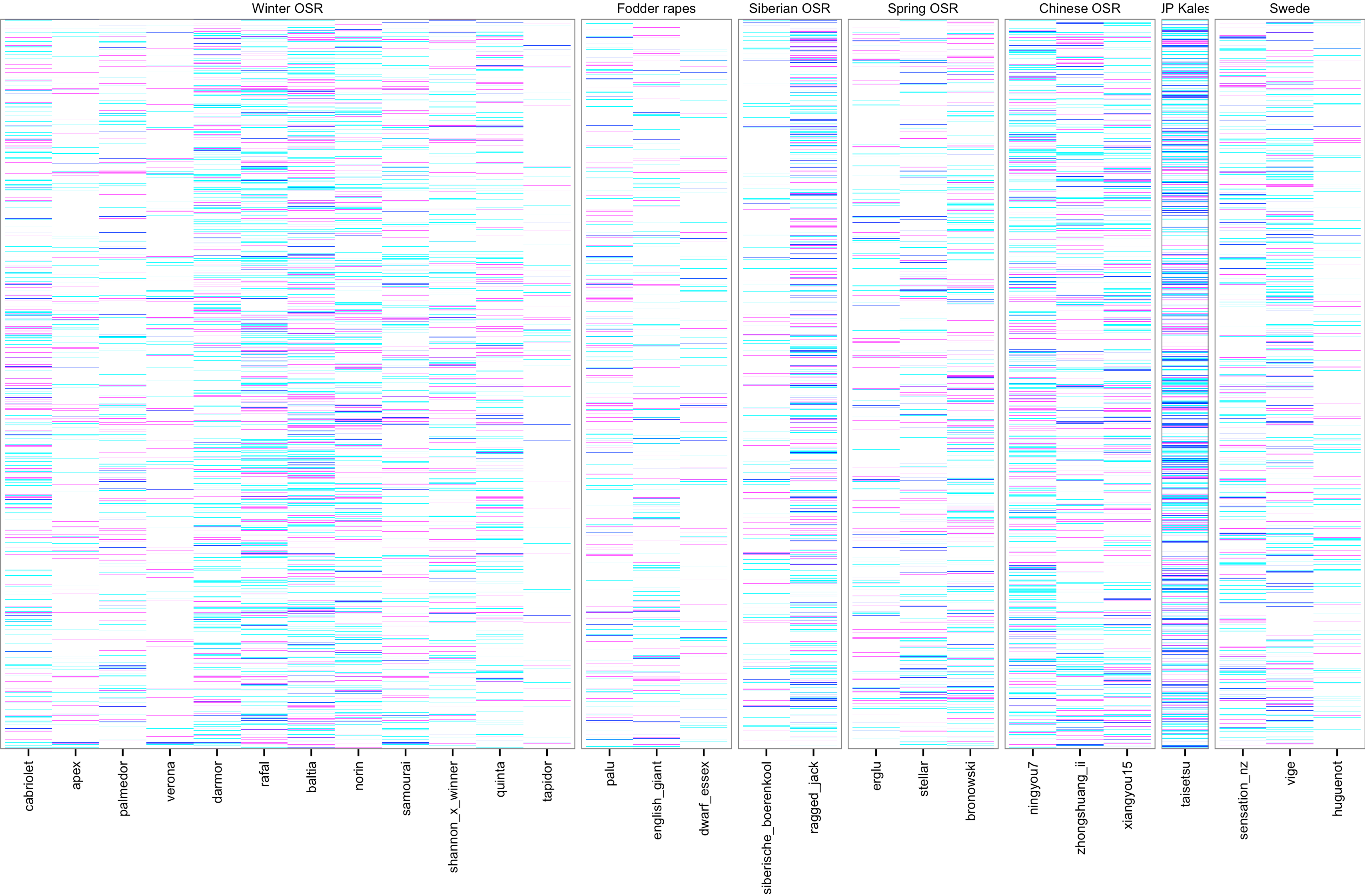

C06

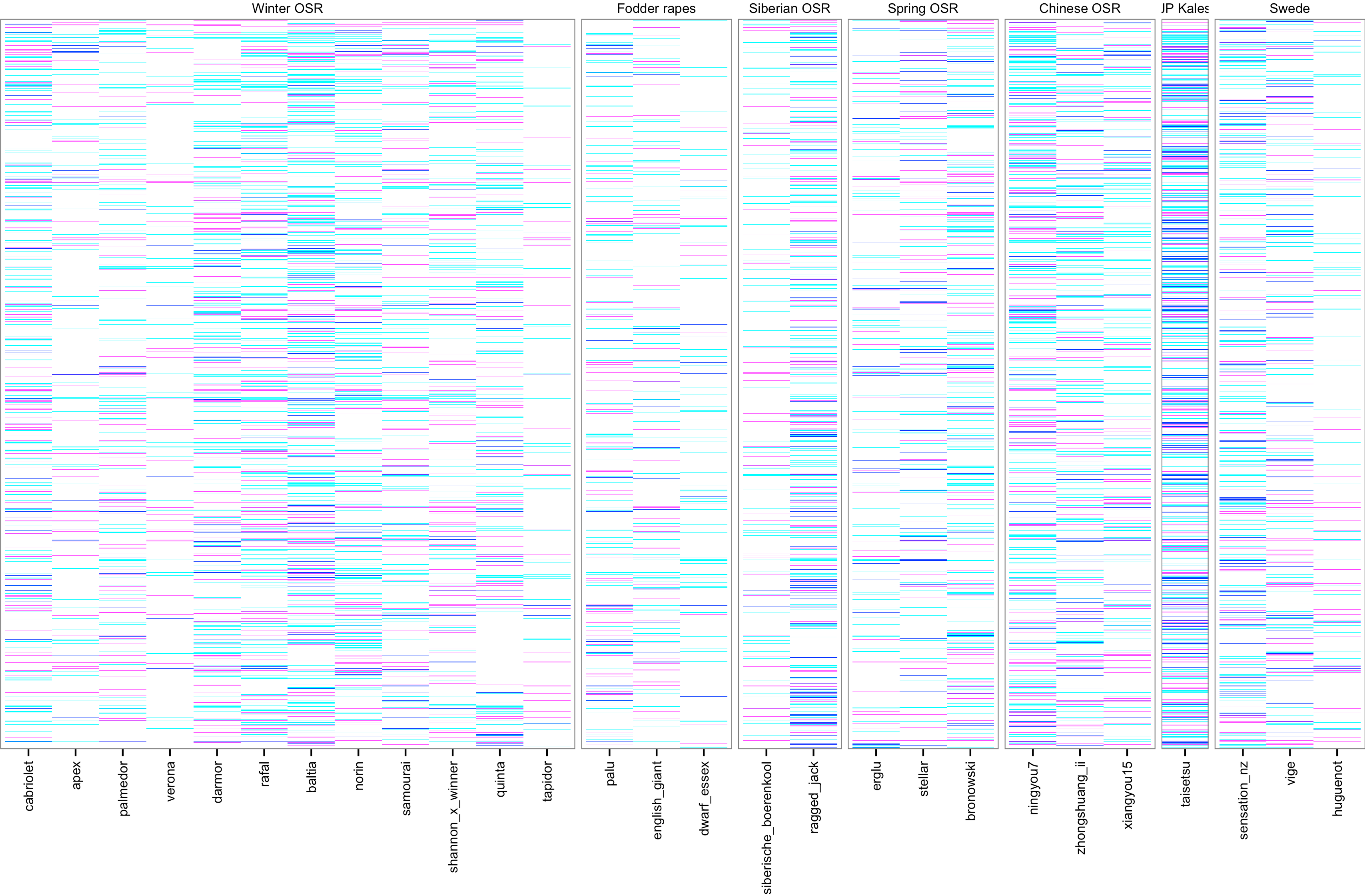

C07

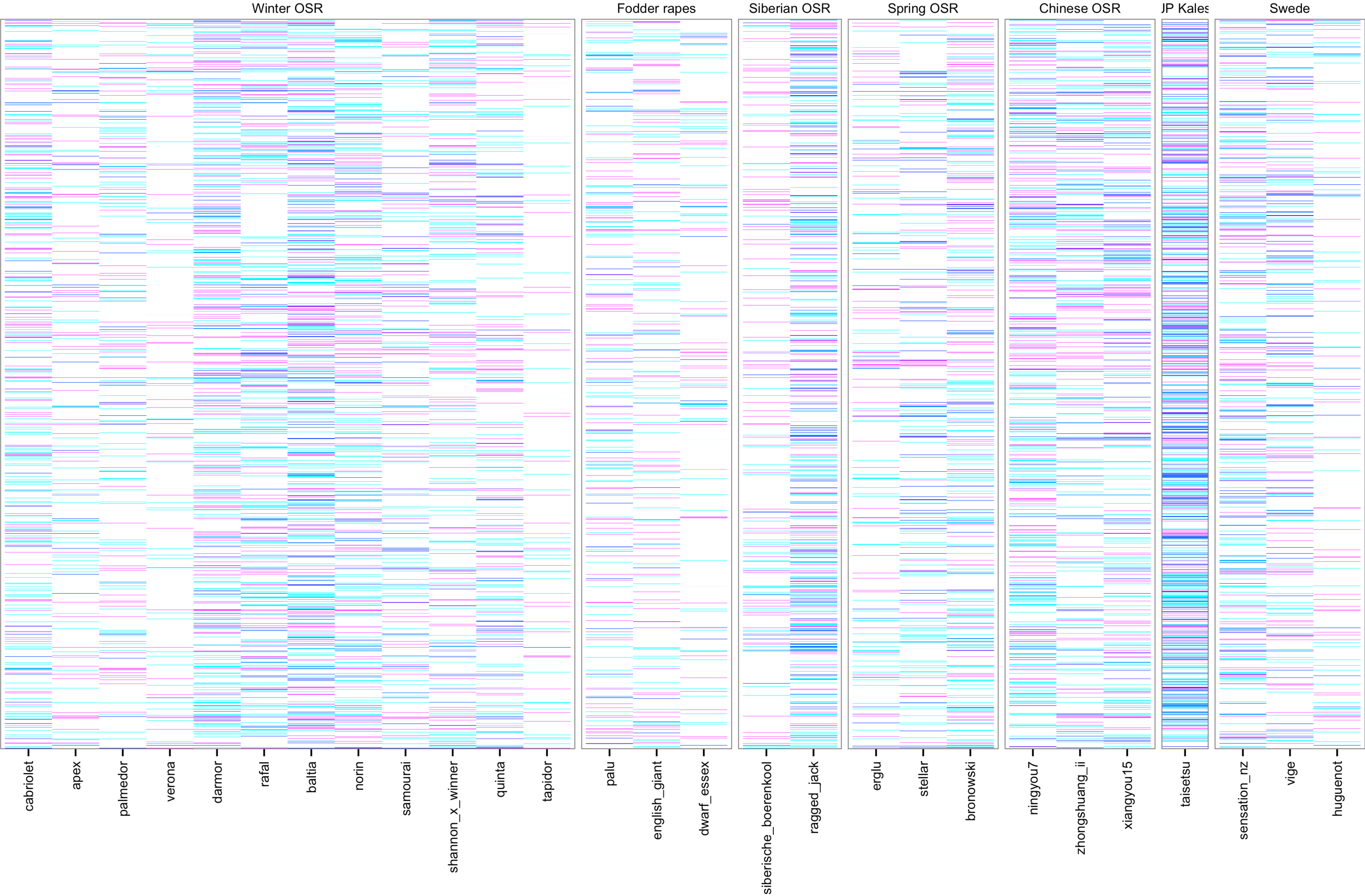

C08

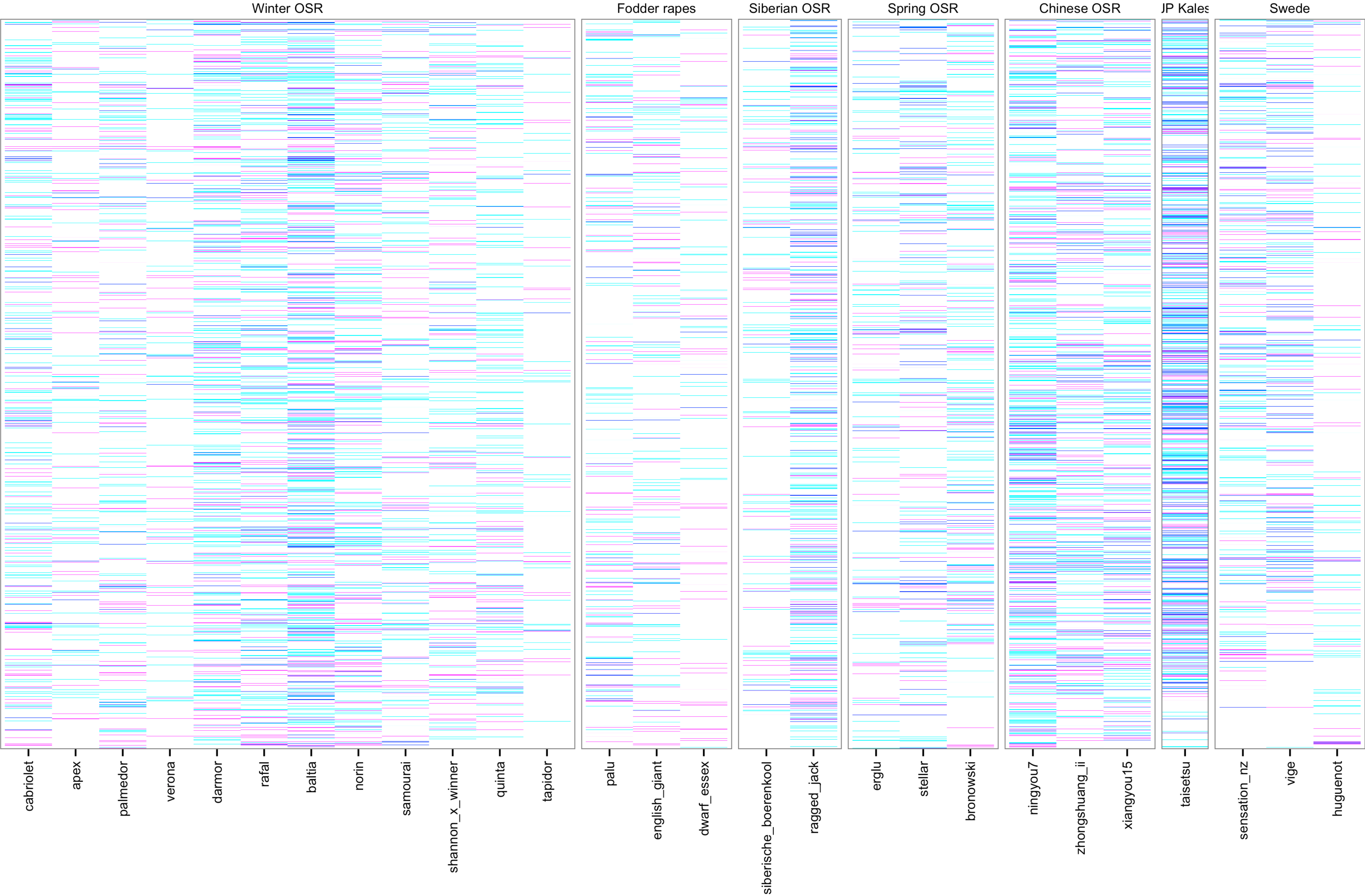

C09

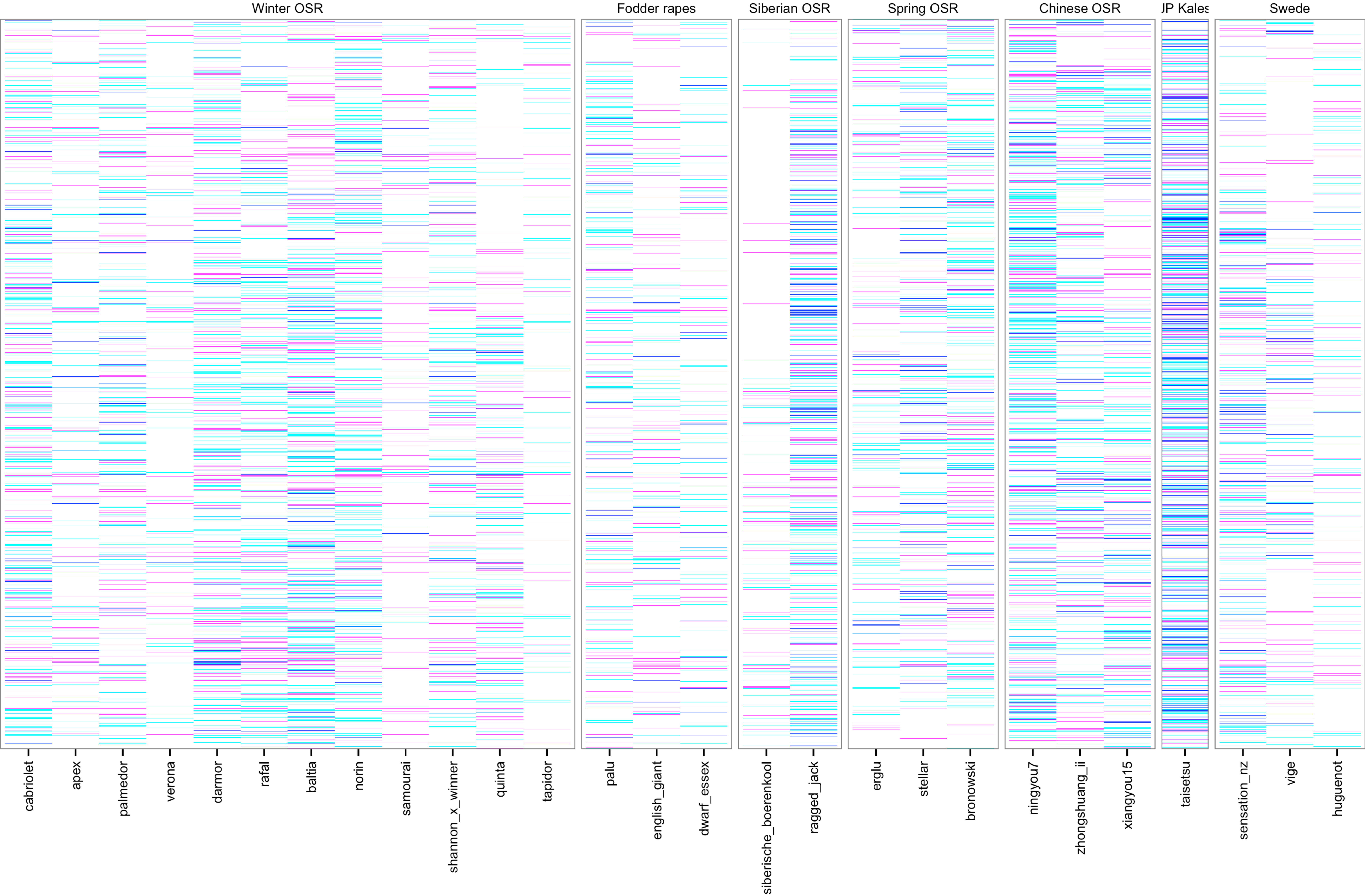

Supplement: Supplementary file 4 — Data S4 Over‐expressing Brassica AB genes. [file PBI-15-594-s008.pdf]

C01

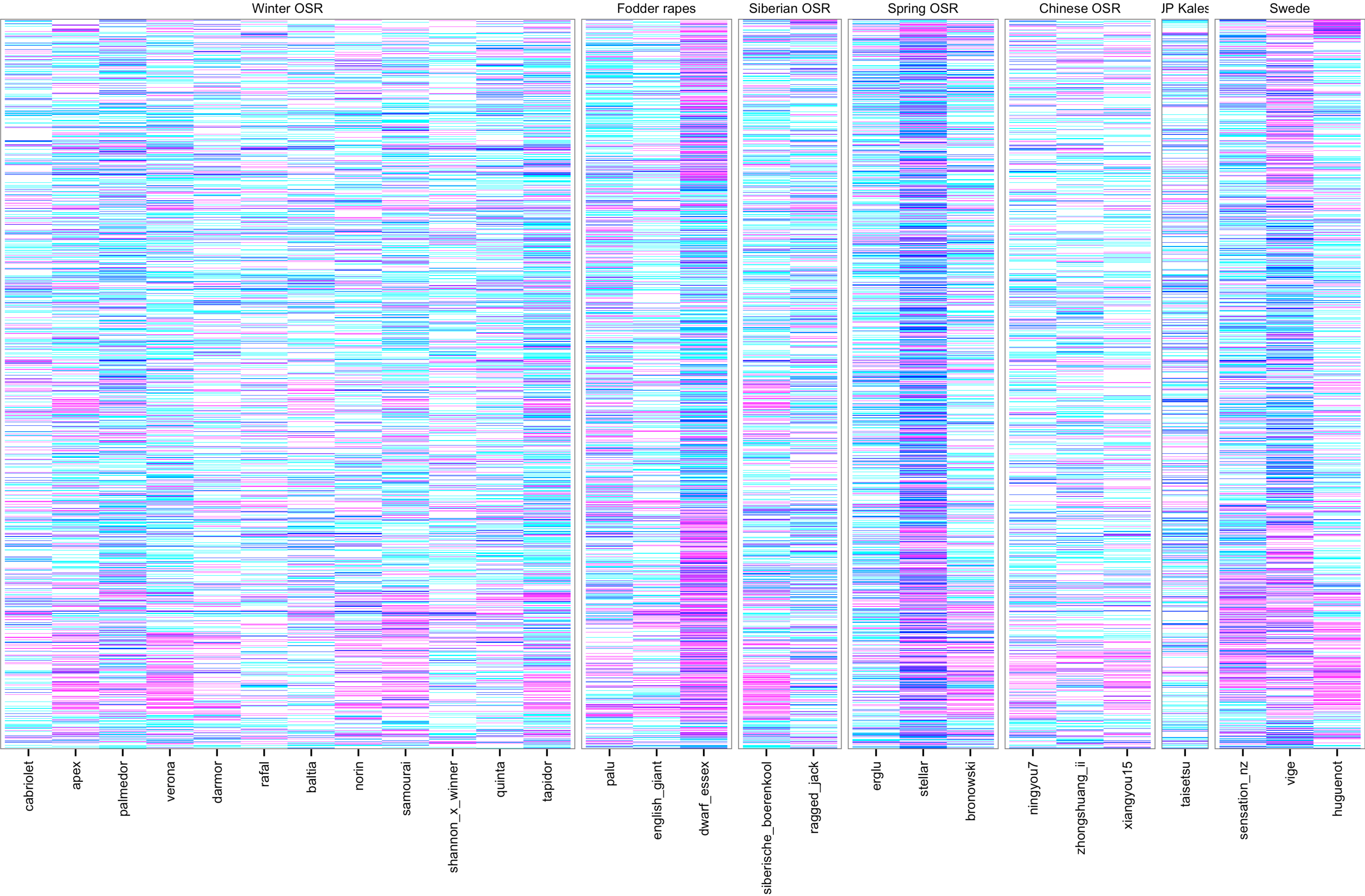

C02

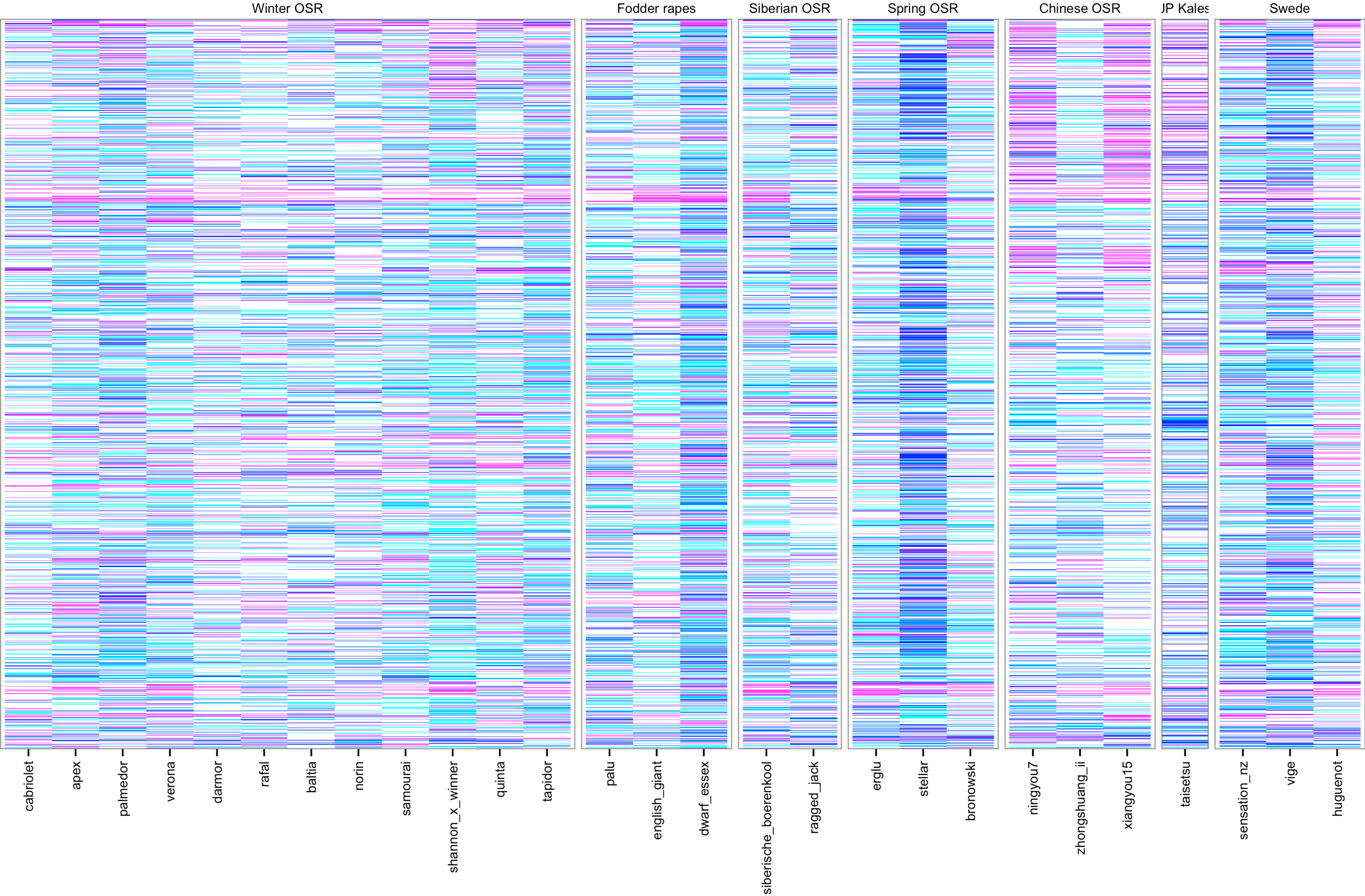

C03

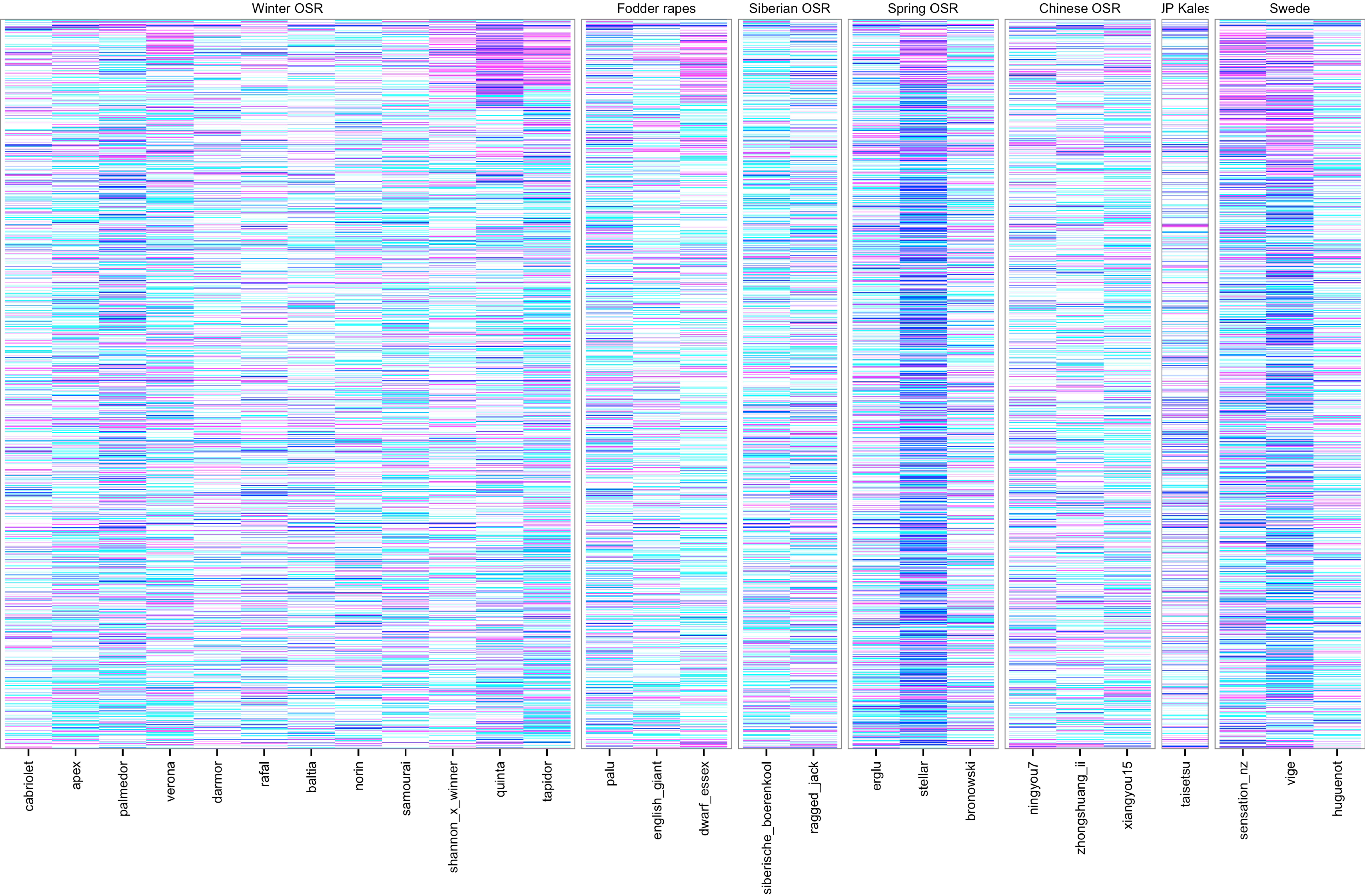

C04

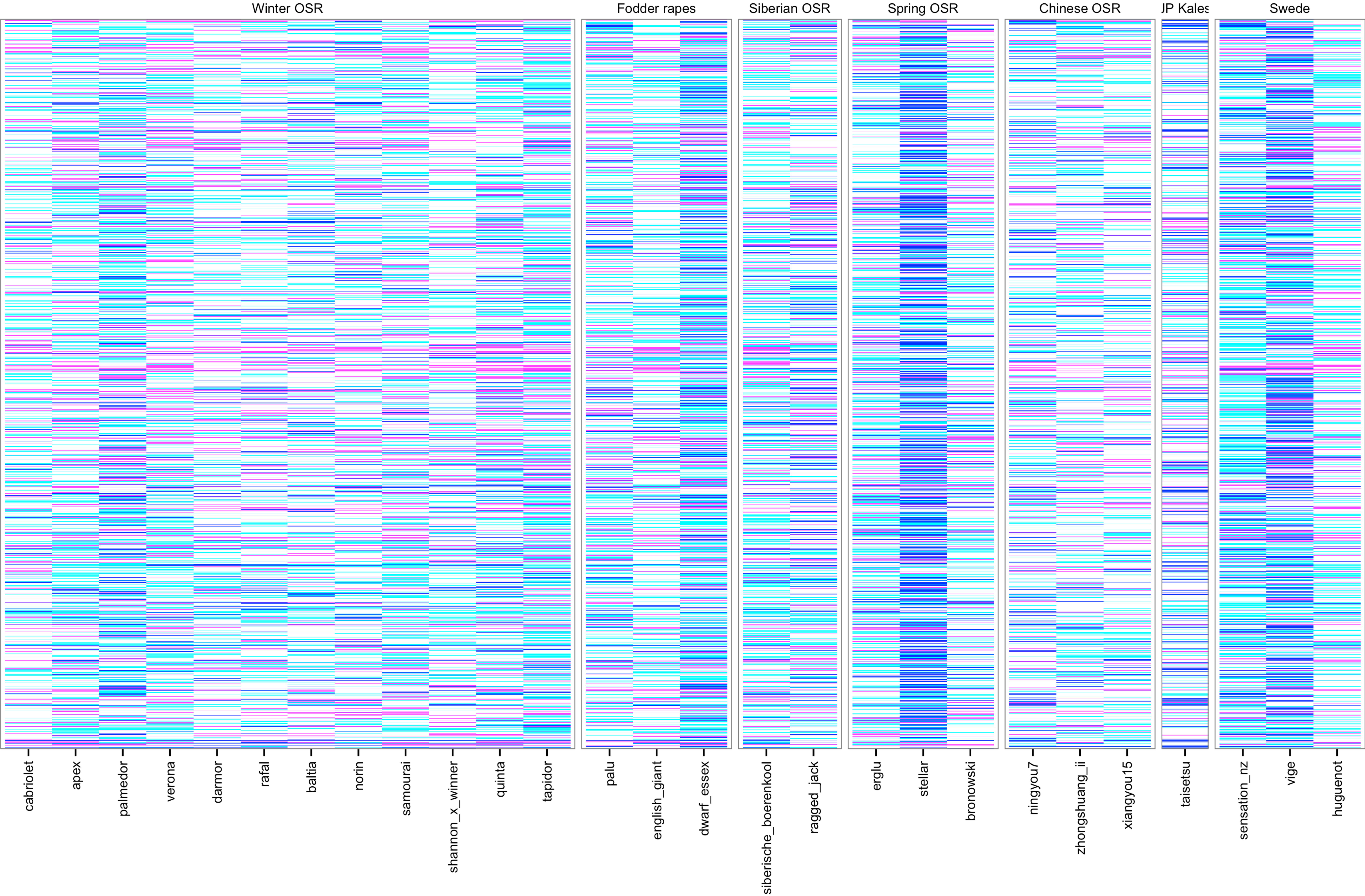

C05

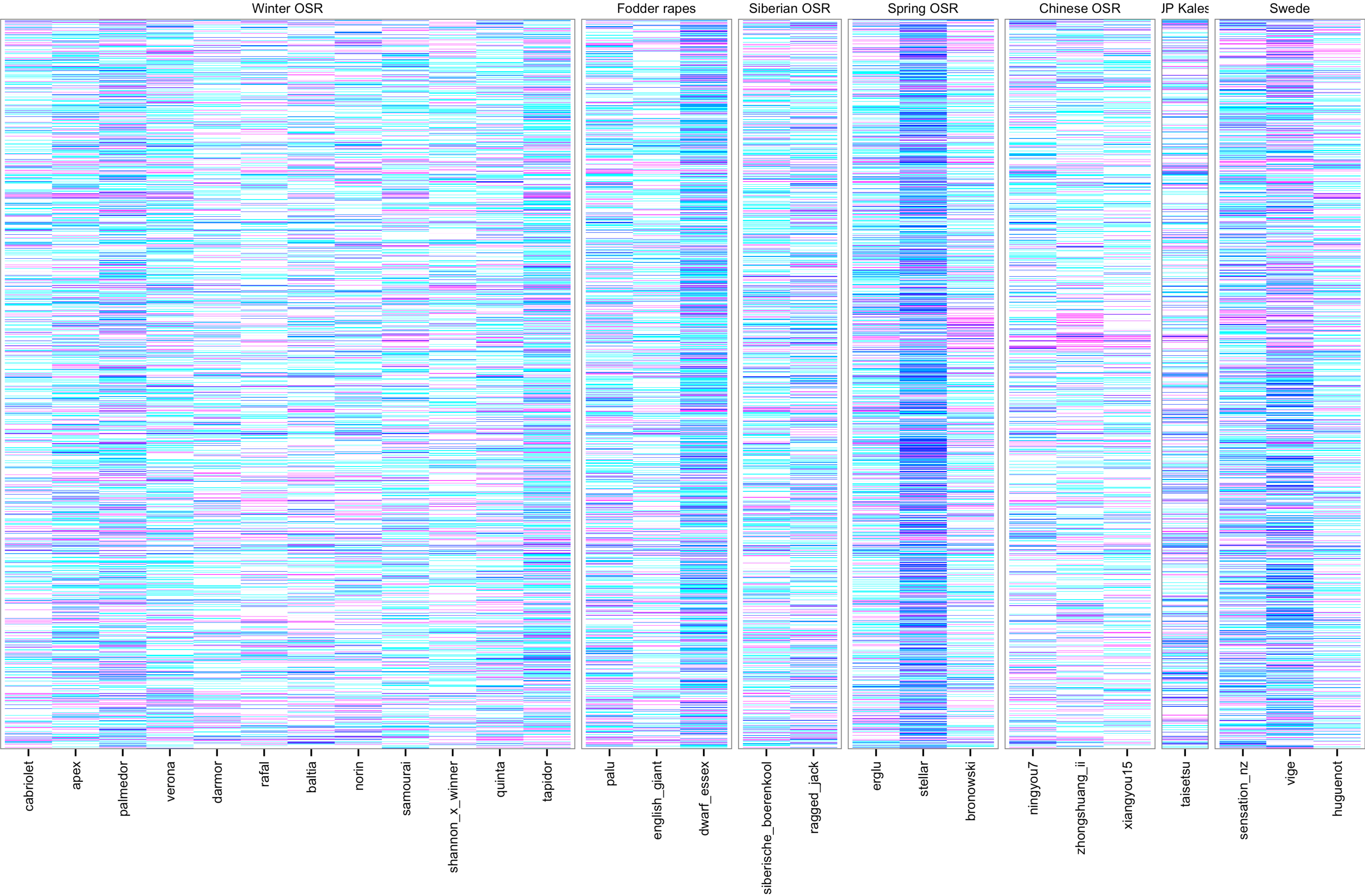

C06

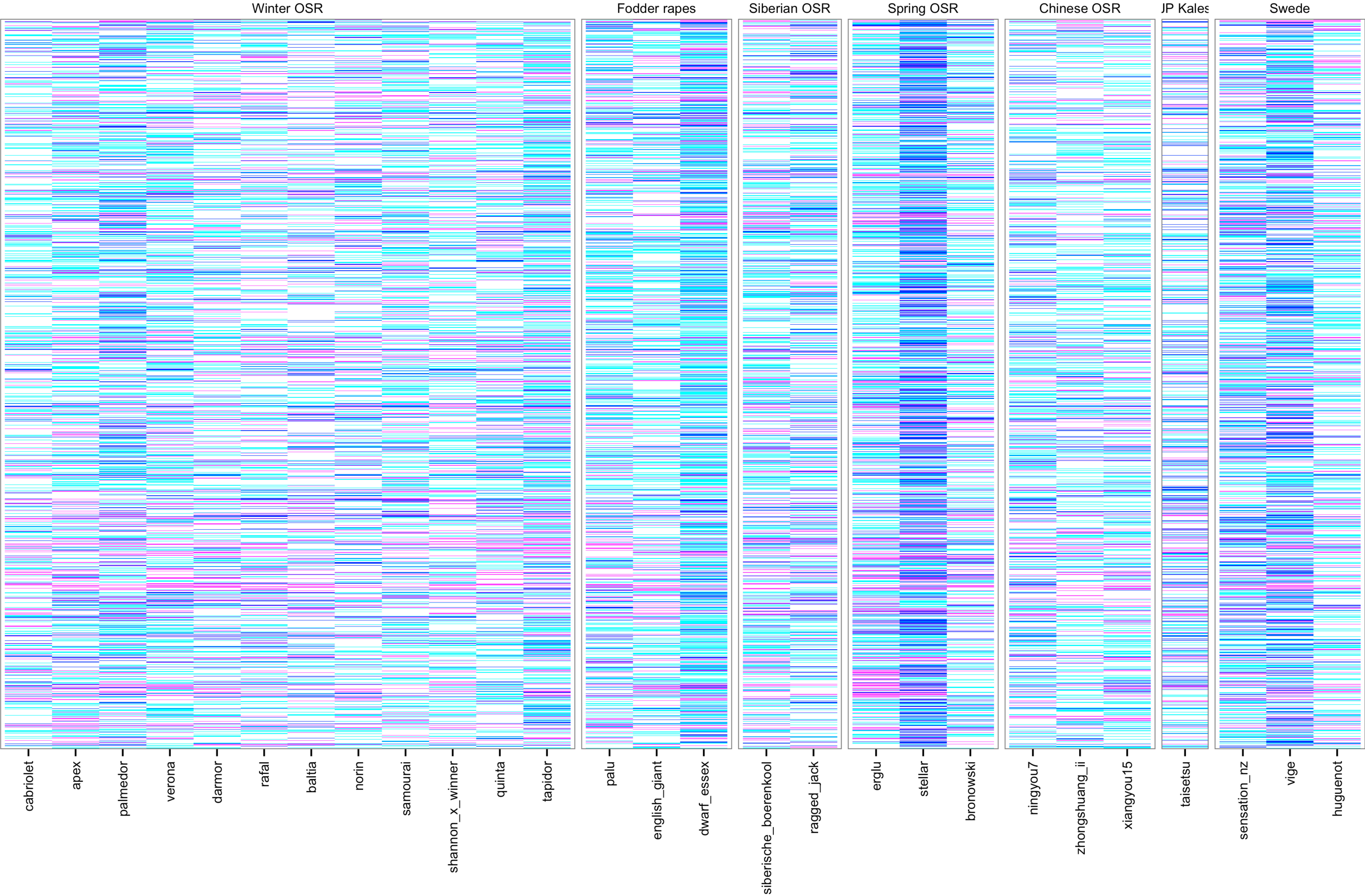

C07

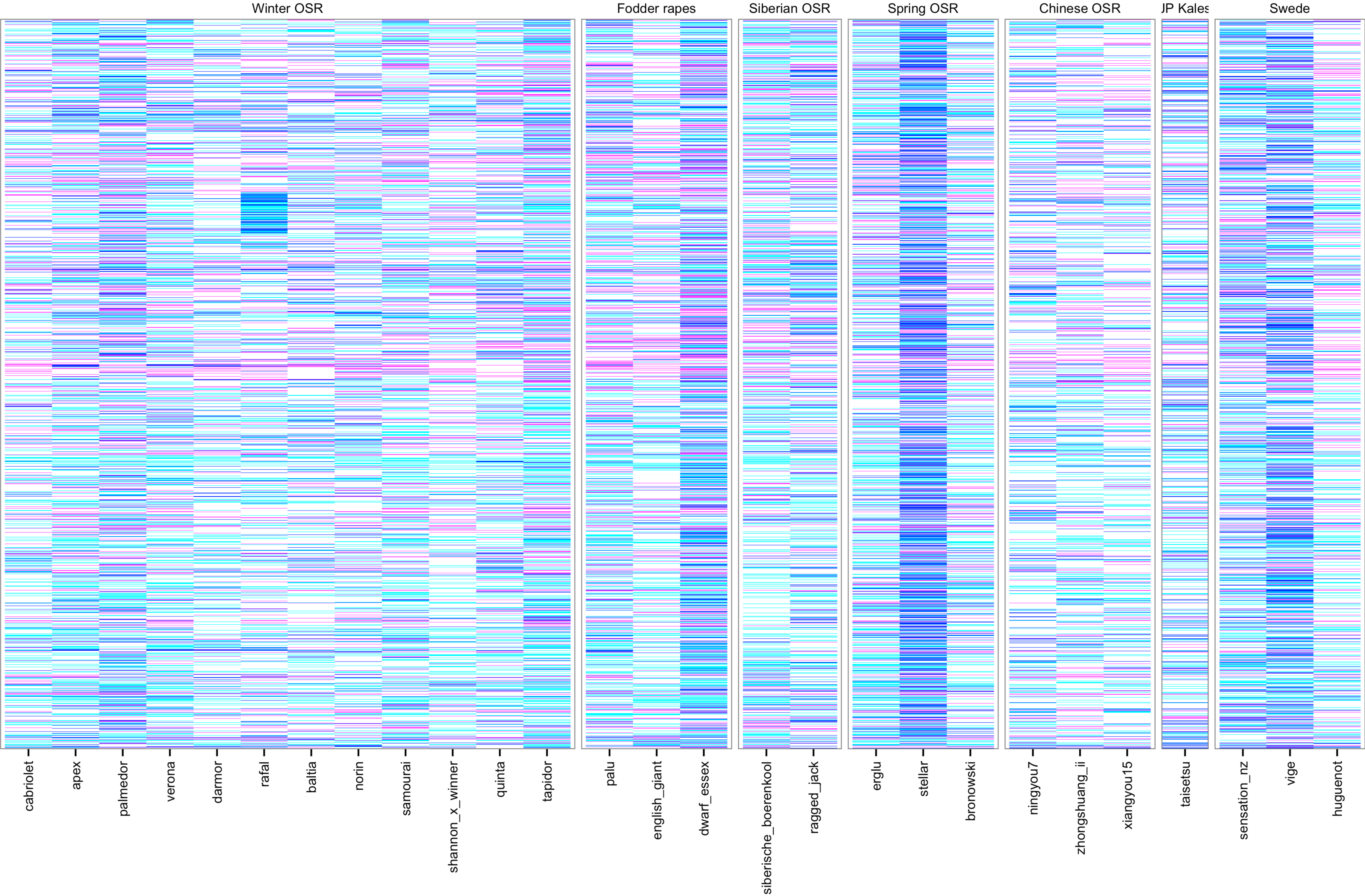

C08

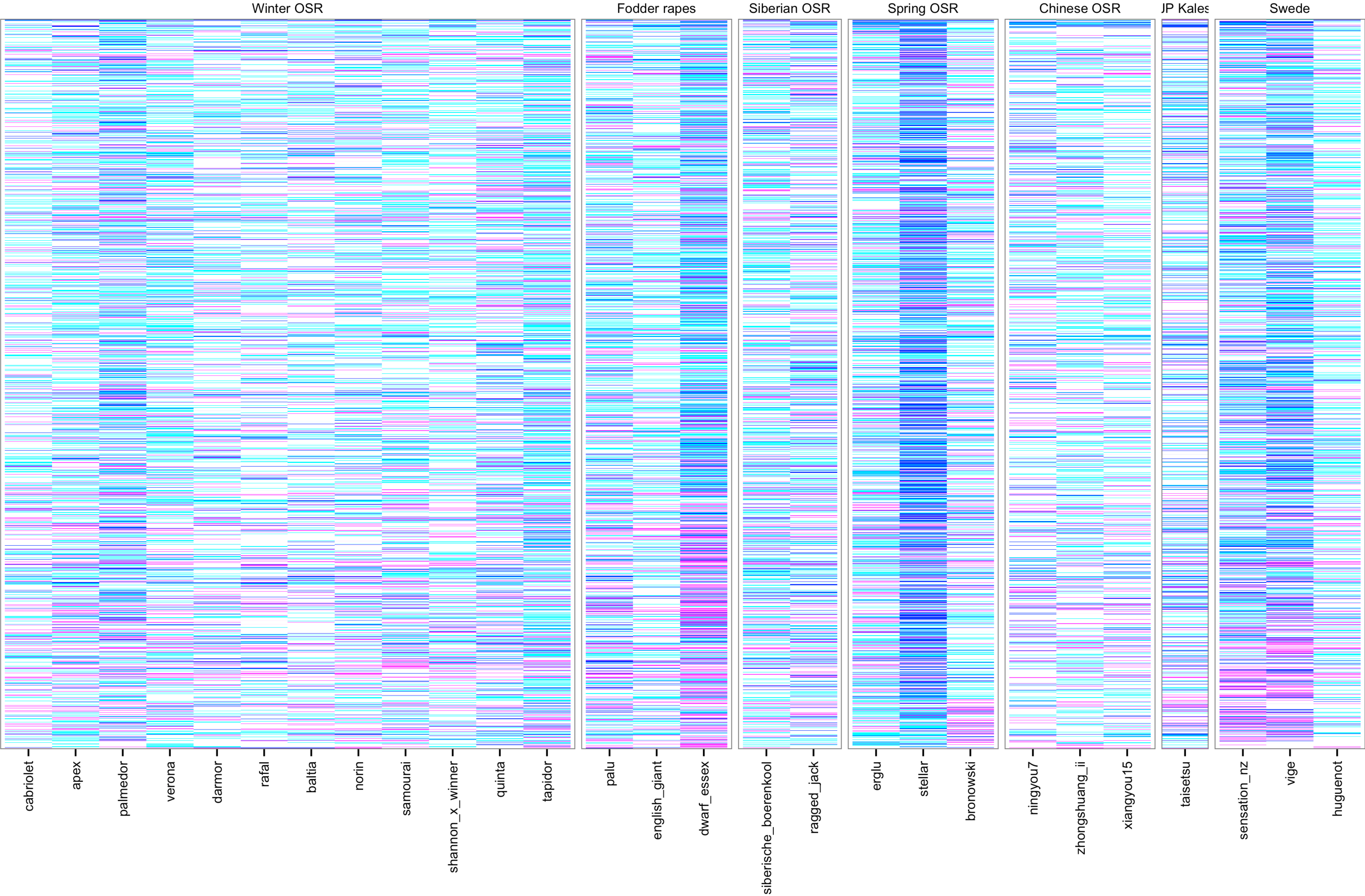

C09

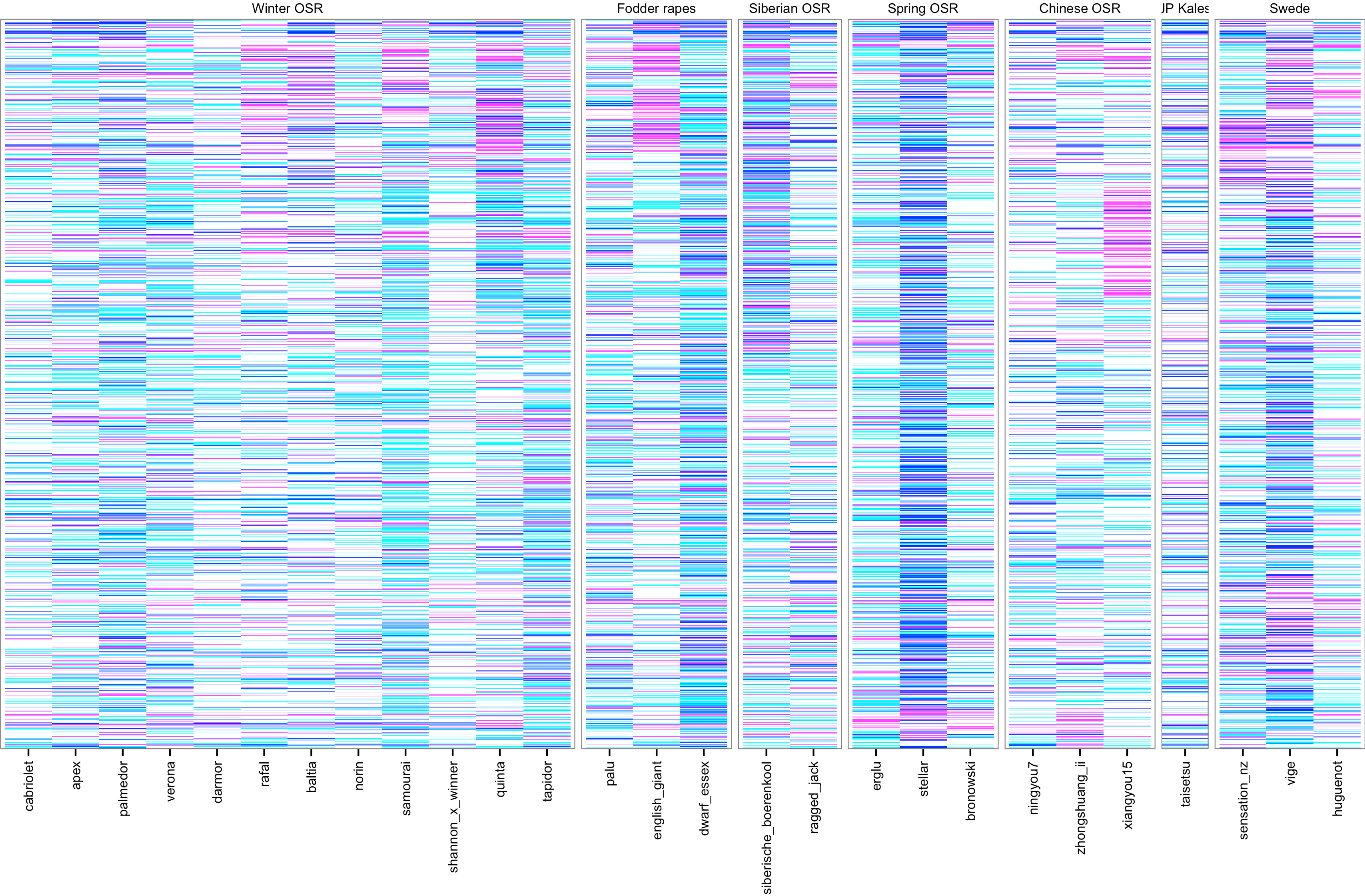

Supplement: Supplementary file 5 — Data S5 Under‐expressing Brassica AB genes. [file PBI-15-594-s001.pdf]
